# A Deep Insight into Perfluorooctanoic Acid Photodegradation Using Metal Ion-Exchanged Zeolites

**DOI:** 10.1021/acsestengg.3c00462

**Published:** 2024-02-15

**Authors:** Lin Qian, Hongying Zhao, Ariette Schierz, Katrin Mackenzie, Anett Georgi

**Affiliations:** † Department of Environmental Engineering, Helmholtz Centre for Environmental Research − UFZ, Permoserstr. 15, D-04318 Leipzig, Germany; ‡ School of Chemical Science and Engineering, Shanghai Key Lab of Chemical Assessment and Sustainability, Key Laboratory of Yangtze River Water Environment, Tongji University, 1239 Siping Road, Shanghai 200092, China

**Keywords:** perfluorooctanoic acid, Fe-zeolites, ion-exchanged zeolites, photochemistry, degradation

## Abstract

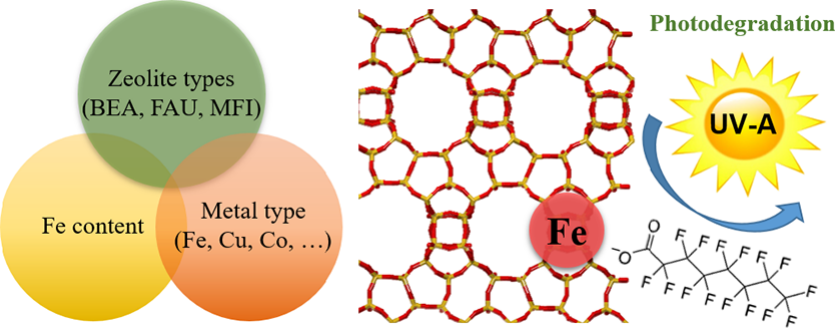

Treating perfluorooctanoic acid (PFOA) in an aqueous environment is problematic due to its low concentration and its high resistance to biological and chemical degradation. To tackle this challenge, combinations of pre-enrichment and photodegradation processes are promising solutions. In this work, we investigated metal ion-exchanged zeolites as adsorbents and photocatalysts for PFOA treatment. Among various transition metal ion-exchanged BEA zeolites, Fe-exchanged BEA (Fe-BEA) zeolites showed significant activity for the photodegradation of PFOA. The isolated iron species in Fe-BEA zeolite are responsible for PFOA photodegradation, whereas other iron species present from excess iron loading in the zeolite will lower its photocatalytic activity. Furthermore, it was proved via size exclusion tests using branched PFOA isomers that the photodegradation of PFOA took place on the internal surface rather than the external surface of Fe-BEA zeolite. Photodegradation of PFOA was also tested to be effective with Fe-exchanged BEA-type zeolites having various SiO_2_/Al_2_O_3_ ratios, but ineffective with FAU-type zeolites. The optimal Fe-BEA zeolite showed a sorption coefficient *K*_d_ of 6.0 × 10^5^ L kg^–1^ at an aqueous phase PFOA concentration of 0.7 μg L^–1^ and a PFOA half-life of 1.8 h under UV-A irradiation. The presented study offers a deeper understanding of the use of metal ion-exchanged zeolites for photodegradation of PFOA.

## 1 Introduction

Per- and polyfluoroalkyl substances (PFAS) are emerging persistent organic pollutants, which have drawn much attention in the last few decades. PFAS have been extensively used in industrial and consumer products, e.g., aqueous film-forming foams (AFFF) for fire-fighting, nonstick cookware sets, semiconductors, and Teflon-related products, due to the oleophobic and hydrophobic properties from the perfluorocarbon chain moieties in PFAS.^1^ However, recent studies have shown that the exposure of PFAS at trace levels may cause adverse effects on human health including developmental toxicity, immune toxicity, and hepatotoxicity.^2−4^ Perfluorooctanoic acid (PFOA) is one of the most important PFAS compounds which are frequently detected globally in surface water, groundwater, soil, sediments, and even in animals and human serums.^5,6^ Although production and application of PFOA were recently restricted, the risk of human exposure due to accumulation in marine systems and contaminations in groundwater used for drinking water production will continue to exist for decades.^7^ PFOA removal is problematic as it is reported to slip through wastewater treatment plants and remain in the treated water.^8^ Additionally, PFOA is resistant to most conventional reduction, oxidation, and biological degradation processes due to the high strength of the C–F bonds (*D*_C–F_ ≈ 485 kJ mol^–1^).^9^ Furthermore, it is a huge challenge to treat diffuse PFOA contaminations in the aqueous environment due to their low concentrations (commonly at ng or μg L^–1^ level).^10^ Therefore, the entry of PFOA into these water streams has to be prevented by means of suitable measures.

Several physicochemical approaches for PFOA destruction (e.g., photochemical, electrochemical, sonochemical, plasma, and radiolytic approaches) have been investigated and established.^11−15^ Among them, the photochemical approach is especially promising, as it has the potential to be directly powered by sunlight. However, the efficiency of all of these degradation approaches is restricted by low PFOA concentrations and complex water matrices. In addition, treatment times are typically on the order of several hours, which is infeasible for large-volume water treatment. In order to tackle this challenge, degradation processes need to be combined with pre-enrichment steps. The ideal solution for PFOA degradation is thus developing a material serving both as adsorbent and photocatalyst, which enables (I) efficient PFOA removal from the large stream of contaminated water by safe adsorption and (II) on-site adsorbent regeneration by photochemical degradation of PFOA. A variety of photocatalysts, for instance, titanium(IV) oxide,^16^ indium(III) oxide,^17^ gallium(III) oxide,^18^ some photocatalyst composite materials,^19,20^ etc., showed adequate activity for PFOA degradation. However, such catalysts usually possess relatively low adsorption capability to PFOA, which limits their application in real water treatment.

One of the few known adsorptive photocatalysts is a composite with activated carbon-supported indium-doped titanate nanotubes (In/TNT@AC) recently developed by Arana Juve et al.^21^ for PFOA photodegradation. The composite material shows good adsorption (>99% in 30 min) and photodegradation (>99% in 4 h) performance to PFOA under optimized conditions (0.1 mg L^–1^ PFOA, 1 g L^–1^ catalyst, UV–C). Tian et al.^22^ designed an iron-doped carbon-modified composite (Fe/TNTs@AC) photocatalyst for the degradation of a set of PFAS. Thirteen PFAS in municipal landfill leachate were removed by >95% within 8 h under UV irradiation with 10 g L^–1^ fresh Fe/TNTs@AC. Xu et al.^23^ demonstrated PFOA photodegradation using an iron (hydr)oxides/carbon sphere (FeO/CS) composite. Trace PFOA can be enriched by FeO/CS from water, and the preconcentrated PFOA can be degraded under simulated solar light. Most of the reported adsorptive photocatalytic degradation approaches toward PFOA are based on carbon composite catalysts. Carbonaceous materials can be modified to possess high specific surface area and strong adsorption affinities to varieties of micropollutants (including PFOA).^24−26^ However, carbon not only has a strong shading effect, but the surface of carbonaceous materials is also susceptible to attack by reactive oxygen species,^27^ which will be inevitably formed during PFOA photodegradation. Studies of other adsorptive photocatalysts with higher stabilities for PFOA degradation are limited.

Besides carbonaceous materials, zeolites are widely used as adsorbents and catalysts in industrial processes, e.g., oil refining and petrochemical industries.^28^ Unlike carbon, zeolites are robust under most oxidative conditions and provide, in addition, a certain transparency to light. Hence, zeolites are suitable candidates as long-lived recyclable catalysts that can adsorb recalcitrant pollutants but maintain the adsorption capacity during the regeneration process in the presence of strong oxidants. Nonetheless, not all types of zeolites are suitable for adsorption of PFOA. Among a few commercially available zeolite types we have screened (Figure S1), i.e., beta zeolites (BEA), faujasite zeolites (FAU), Mobil-type five zeolites (MFI), BEA zeolites were found to be suitable adsorbents for PFOA (single point adsorption coefficients *K*_d_ can reach 10^5^ L kg^–1^ at an equilibrium aqueous phase PFOA concentration (*C*_free_) in the range of 8 to 20 μg L^–1^). BEA zeolites consist of channels with 12 T atoms (T = Si or Al), in the form of zigzag channels (Ø 5.6–5.7 Å) and larger straight channels (Ø 6.6–6.7 Å). The latter can ideally fit linear PFOA molecules, as their effective chain diameter is about 6 Å.^29^ FAU zeolites have slightly wider channel apertures of Ø 7.4 Å, but the main difference is that their pore volume is largely formed by so-called supercages (about Ø 12 Å) resulting from channel crossings, which are obviously less favorable for PFOA adsorption, at least in the concentration range tested here. MFI zeolites, due to their narrower 10 T atom channels (max. Ø 5.6 Å), do not allow efficient uptake of PFOA.

In our previous work, we applied an iron-exchanged BEA zeolite (Fe-BEA35) for PFOA photodegradation.^11^ Fe-BEA35 was found to enrich PFOA effectively from water and facilitate PFOA photodegradation under UV-A light for in situ regeneration. We demonstrated that the ligand-to-metal charge transfer under UV-A irradiation plays a major role in the photodegradation of PFOA (detailed mechanism in the SI, Text S3). To the best of our knowledge, commercially available Fe-BEA35 is the only zeolite material tested for the photodegradation of PFOA so far. It is thus of great interest to understand further the process and address the questions: Is the Fe-BEA35 with 1.3 wt % Fe content the best zeolite for PFOA photodegradation, or is there an optimal iron content? What are the contributions and influences of various iron species in Fe-BEA35 during PFOA photodegradation? What are the roles of the internal and external surfaces of the zeolite particles during PFOA photodegradation? Is iron the most active transition metal for PFOA photodegradation, or are there any other alternative transition metals for this process? Can other types of iron-exchanged zeolites be used for PFOA photodegradation? In this work, we have performed comprehensive material characterization for iron-exchanged BEA zeolites with various metal contents and correlated the content of various iron species with PFOA photodegradation activities. In addition, several transition metal ion-exchanged BEA zeolites and other types of iron-exchanged zeolites were synthesized and characterized, and their performance in PFOA photodegradation was compared. The degradation intermediates were investigated in detail, and the roles of the internal and external zeolite surfaces for catalysis were verified. Overall, we provide a deeper insight into PFOA photodegradation by ion-exchanged zeolites, which can inspire future applications for the treatment of PFAS and other recalcitrant trace-level contaminants.

## 2 Experimental Section

Detailed information about materials, chemicals, catalyst preparation, photochemical degradation processes, and analyses are described in the Supporting Information (SI, Text S1). For a regular photochemical PFOA degradation, 30 mL of aqueous PFOA solution (48 μM) was mixed with metal ion-exchanged zeolites. Photodegradation was started after an initial 24 h dark period for achieving adsorption equilibrium (Figure S15 in the SI). In order to follow PFOA degradation, the total PFOA concentrations were determined by exhaustive extraction of the zeolite. The UV-A mercury lamp was placed beneath the quartz reactor. The distance between the bottom of the quartz reactor and the UV lamp window was 20 mm. The spectral curve of the UV-A lamp is shown in Figure S2. The photon flux was measured by ferrioxalate actinometry to be 4.47 × 10^–6^ mol s^–1^.

## 3 Results and Discussion

### 3.1 Characterization of Iron-Exchanged BEA35

The theoretical maximum cation exchange capacity of zeolites can be estimated on the basis of the Al content, assuming that each Al atom in the zeolite structure creates a negatively charged site and attracts protons when suspended in water. These protons are associated with the Bro̷nsted acidity of zeolites and can be exchanged with certain metal cations. The zeolite acidity is consequently reduced after cation exchange. When exchanging H^+^ in BEA35 zeolite by Fe^2+^, the theoretical maximum capacity of iron in the BEA35 is estimated to be 4.8 wt %, assuming Fe^2+^ to be complexed by one (AlO_4_)^−^ site. In this study, four Fe-BEA35 zeolites were prepared by ion exchange with different iron(II) concentrations. The total iron amounts in the zeolites determined by XRF are shown in Table S1. The four Fe-BEA35 samples were named according to their iron contents in wt %, i.e., 0.52 Fe-BEA35, 1.26 Fe-BEA35, 1.61 Fe-BEA35, and 2.36 Fe-BEA35, which follow the trend in dosed iron concentration in the exchange process. The ion-uptake efficiency during the ion-exchange process is determined by eq 2: 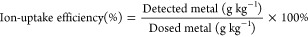
1

It is clear that the ion-uptake efficiency decreases with an increasing amount of dosed iron(II) salt as exchangeable sites get saturated. Even assuming that all detected iron in the zeolite is present as isolated iron species on ion-exchange sites, the theoretical maximum uptake capacity of BEA35 was not reached in all samples. Nevertheless, the Fe/Al molar ratios increase from 0.10 to 0.48 with increasing Fe content. This could mean that not all ion-exchange sites are accessible for iron ions or that the coordination of iron ions involves more than one (AlO_4_)^−^ site, which also strongly depends on their proximity (note that there is only one Al atom for about 18 Si atoms in BEA35).

**Figure 1:**
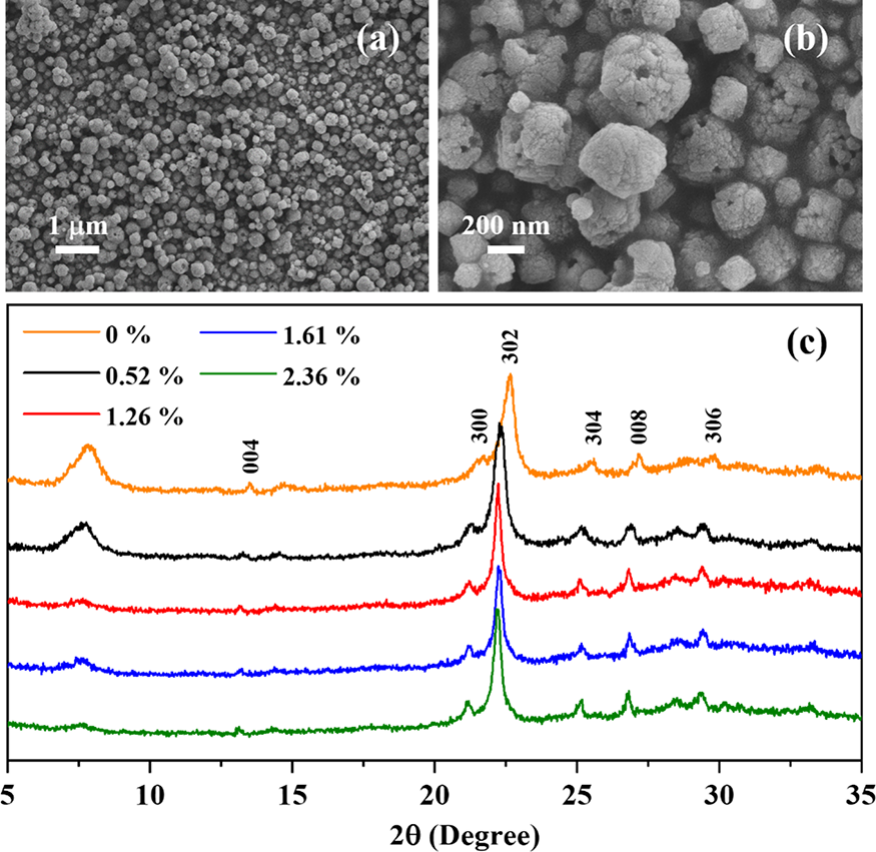
(a, b) Scanning electron microscope (SEM) images of BEA35 zeolites; (c) X-ray powder diffraction pattern of iron-exchanged BEA35 zeolites with various iron contents.

The morphology of the Fe-BEA35 samples was characterized by means of scanning electron microscopy (SEM) as shown in Figures 1a,b and S3. As seen, the BEA35 and all four Fe-BEA35 zeolites comprise joined zeolite crystallites without sharp edges, and the diameter of the majority of particles (90%) is about 0.2–0.4 μm (Figure S3). The ion-exchange process is proven not to affect the morphology of BEA35 particles. X-ray diffraction (XRD) analyses were performed to evaluate the zeolites’ crystallographic structure after the ion-exchange process (Figure 1c). The two major diffraction peaks around 2θ = 7.5° and 2θ = 22.6° are assigned to the faulted structure due to the coexistence of two polymorphs in BEA-type zeolites and the expansion/contraction of the BEA structure, respectively. All diffraction peaks shown by the pristine BEA35 are observed in the diffraction patterns of the four Fe-BEA35 zeolites at nearby positions. Thus, in general, the ion-exchange process does not significantly change the zeolite crystallographic structure. Nonetheless, the peak around 2θ = 7.5° shrank with increasing iron content. In addition, it can be observed that some diffraction peaks of Fe-BEA35 are shifted to smaller angles, which usually indicates the expansion of the zeolite lattice. The *d*_302_ spacing increases from 3.933 (BEA35, 2θ = 22.59°) to 3.983 Å (0.52 Fe-BEA35, 2θ = 22.32°), 3.996 Å (1.26 Fe-BEA35, 2θ = 22.23°), 3.994 Å (1.61 Fe-BEA35, 2θ = 22.24°), and 4.003 Å (2.36 Fe-BEA35, 2θ = 22.19°), with the increasing iron contents in the Fe-BEA35 zeolites.

### 3.2 Photodegradation of PFOA Using Fe-BEA35

**Figure 2:**
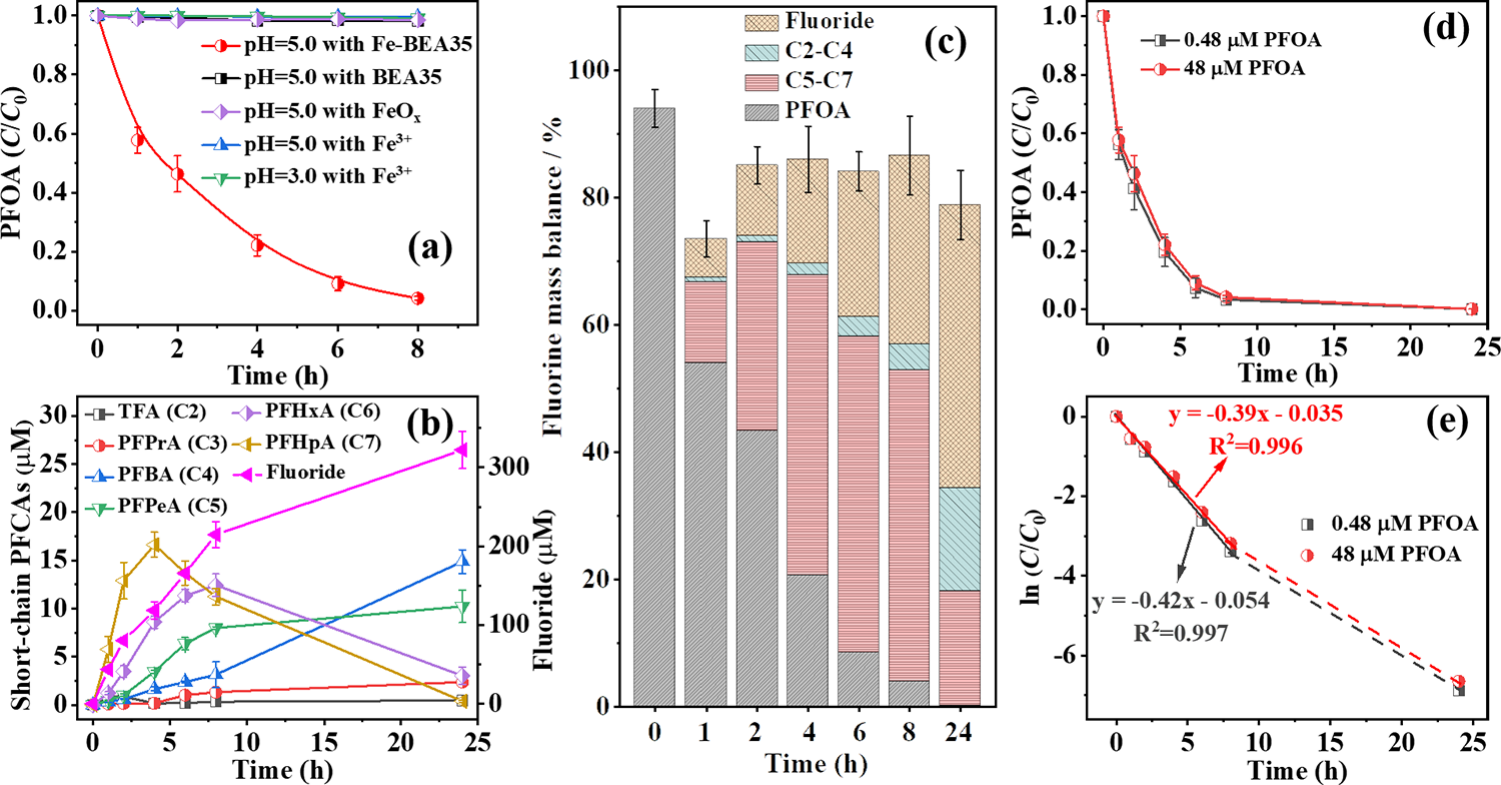
(a) Comparison of PFOA photodegradation in the presence of (i) ferric ions, (ii) iron oxide nanoparticles, and (iii) BEA35 and (iv) 1.26 Fe-BEA35. *C*_0,PFOA_ = 48 μM, *C*_0,Fe_^3+^ = 200 μM, *C*_iron(III) oxide_ = 0.1 g L^–1^, and *C*_Fe-BEA35_ = 1 g L^–1^, where applied, pH_0_ = 5.0 or 3.0; (b) formation of short-chain PFCA intermediates and fluoride, (c) fluorine mass balance during photochemical degradation of PFOA; (d) kinetics of photodegradation of PFOA using Fe-BEA35 at two different initial PFOA concentrations; and (e) first-order-kinetics plots of PFOA degradation with linear regression for the period up to 95% turnover. 1 g L^–1^ 1.26 Fe-BEA35, pH_0_ = 5, *C*_0,PFOA_ = 0.48 or 48 μM (0.2 or 20 mg L^–1^). Error ranges stand for the standard deviations of the results from triplicate assays in (a), (b), and (d). The cumulative error is shown in (b). Lines in (a–d) serve as guides for the eye.

First, we verified the effect of BEA35 zeolite, iron(III) oxide nanoparticles, and dissolved ferric ions on the degradation of PFOA under UV-A in comparison to the iron-exchanged zeolite Fe-BEA35 with moderate iron content (1.26 wt %). Photodegradation of PFOA was performed under the same conditions (i.e., 48 μM PFOA and UV-A irradiation) in the presence of Fe-BEA35, BEA35, ferric ions at different pH values, and iron(III) oxide nanoparticles as shown in Figure 2a. During all these experiments, almost no PFOA degradation was observed within 8 h except in the presence of 1.26 Fe-BEA35 (1.26 wt % Fe in zeolite). This indicates that (i) PFOA photodegradation under UV-A is negligible; (ii) BEA35 zeolite cannot contribute to PFOA photodegradation; (iii) complexes either between ferric ions and PFOA or between iron(III) oxide and PFOA cannot be excited under UV-A irradiation, or such complex formation is insignificant. However, in the presence of 1.26 Fe-BEA35, up to 95% of the initial PFOA was degraded within 8 h at pH 5; 99.9% degradation and 44% defluorination ratio was achieved within 24 h.

The intermediates produced during the photodegradation of PFOA were detected and quantified (Figure 2b). Perfluorocarboxylic acids (PFCAs) and fluoride are the major products. The concentrations of PFCAs with 7 and 6 C-atoms, i.e., C7 and C6 achieved maxima at 4 and 8 h, respectively, and decreased thereafter, while the concentration of the C5, C4, C3, and C2 acids increased continuously throughout the whole reaction time. According to the pattern of intermediates, it can be deduced that the photodegradation of PFOA first yields C7, which is decomposed stepwise to shorter-chain PFCAs. Figure 2c shows the fluorine mass balance during the photodegradation of PFOA in which the fluorine-containing compounds are classified into four groups: the remaining PFOA, C5 to C7 PFCAs, C2–C4 PFCAs, and fluoride. The 0 h mass balance represents the fluorine detected as PFOA in the zeolite suspension by acetonitrile extraction before the start of irradiation. The 24 h mass balance represents the fluorine detected directly as fluoride and that was still bound in the short-chain C2 to C4 PFCAs, analyzed in the aqueous phase by IC, and C5 to C7 PFCAs and PFOA by acetonitrile extraction. The initial total recovery of fluorine (i.e., as PFOA) was (94 ± 3) % by acetonitrile extraction (see Section 3.4). After 24 h irradiation, the final recovery of fluorine (79 ± 5) %, which consists mainly of fluoride, C5 to C7 PFCAs, and C2 to C4 PFCAs. The incomplete fluorine mass recovery may be due to some undetected fluorine-containing byproducts and/or byproducts strongly bound to the zeolite. A complete mineralization of PFOA cannot be achieved in this Fe-zeolite UV-A system as Fe-zeolite possesses limited adsorption affinity toward the short-chain PFCAs produced (Figure S4). During the process of PFCAs adsorption on zeolite, two major driving forces are considered, i.e., hydrophobic effect and electrostatic interaction. The adsorption benefits from the hydrophobic effect as the nonpolar perfluoroalkyl part of PFCAs finds an appropriate adsorption environment in the narrow zeolite channels, whereas the electrostatic interactions between the negatively charged zeolite surface and the headgroup of PFCAs counteract the adsorption. The limited adsorption of short-chain PFCAs to zeolite can be explained by the reduced hydrophobic effect due to their decreased carbon fluorine chain lengths. Nevertheless, conversion of PFOA into the practically nonadsorbing shorter-chain acids is already coupled with the regeneration of the zeolite adsorption function, while complete mineralization of residual byproducts can be realized in the regeneration solution by post-treatment with UV/persulfate as we proved in our previous work.^11,30^ Applying UV/persulfate treatment as the post-treatment step after initial selective adsorption of PFOA at the same time mitigates the well-known problem of severe parasitic consumption of sulfate radicals by water matrix components such as chloride.^41^

The adsorption of PFCAs to the zeolite is a precondition for photochemical degradation. The fractions of freely dissolved PFCAs (*X*_free_) and adsorbed PFCAs (*X*_sorb_) can be described by eqs 2 and 3. The loading *q* and the adsorption coefficient *K*_d_ are calculated by eqs 4 and 5:
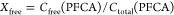
2
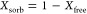
3
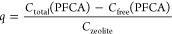
4
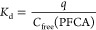
5

Although the adsorption of PFCAs to the zeolite is a precondition, not all adsorbed PFCAs are directly available for photodegradation. Taking PFOA as an example, only ferric-ion-complexed PFOA can be converted during UV irradiation, as mentioned in Section 3.1. Two adsorptive states of PFOA are present in the zeolite channel (Figure S5): complexed PFOA (i.e., specifically adsorbed PFOA at ferric ions) and nonspecifically adsorbed PFOA. The complexed PFOA is characterized by having its carboxylic group in the close vicinity to the ferric ions, which enables the ligand-to-metal charge transfer upon irradiation, while the nonspecifically adsorbed PFOA has less or no chance for charge transfer, as its carboxylic group is not able to interact with the ferric ions. The ratios of complexed and nonspecifically adsorbed PFOA vary under different conditions. For instance, PFOA is predominantly present in the adsorbed state on the zeolite from pH 3 to pH 7, yet limited photodegradation of PFOA is observed at pH 7 (Figure S6), possibly due to the shifted ratio of complexed PFOA to nonspecifically adsorbed PFOA and the change of speciation of the iron complex, as reported in our previous work.^11,30^ There we also derived a rate law suggesting pseudo-first-order degradation of PFOA (eq 6) with *X*_complex_ as fraction of PFOA in reactive complexes (eq 7), which is a certain part of *X*_sorb_:
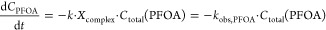
6
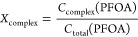
7

The overall degree of sorption *X*_sorb_ can be obtained experimentally, but the *X*_complex_ (≤*X*_sorb_) cannot be determined easily. Equation 6 is valid under the precondition that all equilibria involving PFOA, i.e., its distribution between the freely dissolved, nonspecifically sorbed, and complexed PFOA, are fast compared to its photodegradation reaction and that the equilibrium constants are independent of PFOA concentration. As can be seen in Figure 2e, the pseudo-first-order kinetics model fits well to the initial reaction period (0–8 h) with a PFOA degradation degree of 0–95%, while the PFOA degradation slightly slows down at higher turnover (95–99.9%). In order to exclude the possibility of a slightly decreased PFOA degradation rate caused by the lower PFOA concentration at higher turnover, we decreased the initial PFOA concentration by 2 orders of magnitude, i.e., to 0.2 mg L^–1^. As shown in Figure 2d,e, almost identical PFOA degradation kinetics under these two conditions were observed (initial PFOA concentration at 48 and 0.48 μM). Therefore, we can also expect adequate photodegradation activities of this approach with trace PFOA concentrations. Pseudo-first-order kinetics with the same rate constant of 0.40 ± 0.02 min^–1^ applies to the degradation of PFOA in the concentration range tested, from 20 mg L^–1^ to 7 ng L^–1^.

### 3.3 Influence of Iron Speciation in Zeolite on Photodegradation of PFOA

**Figure 3:**
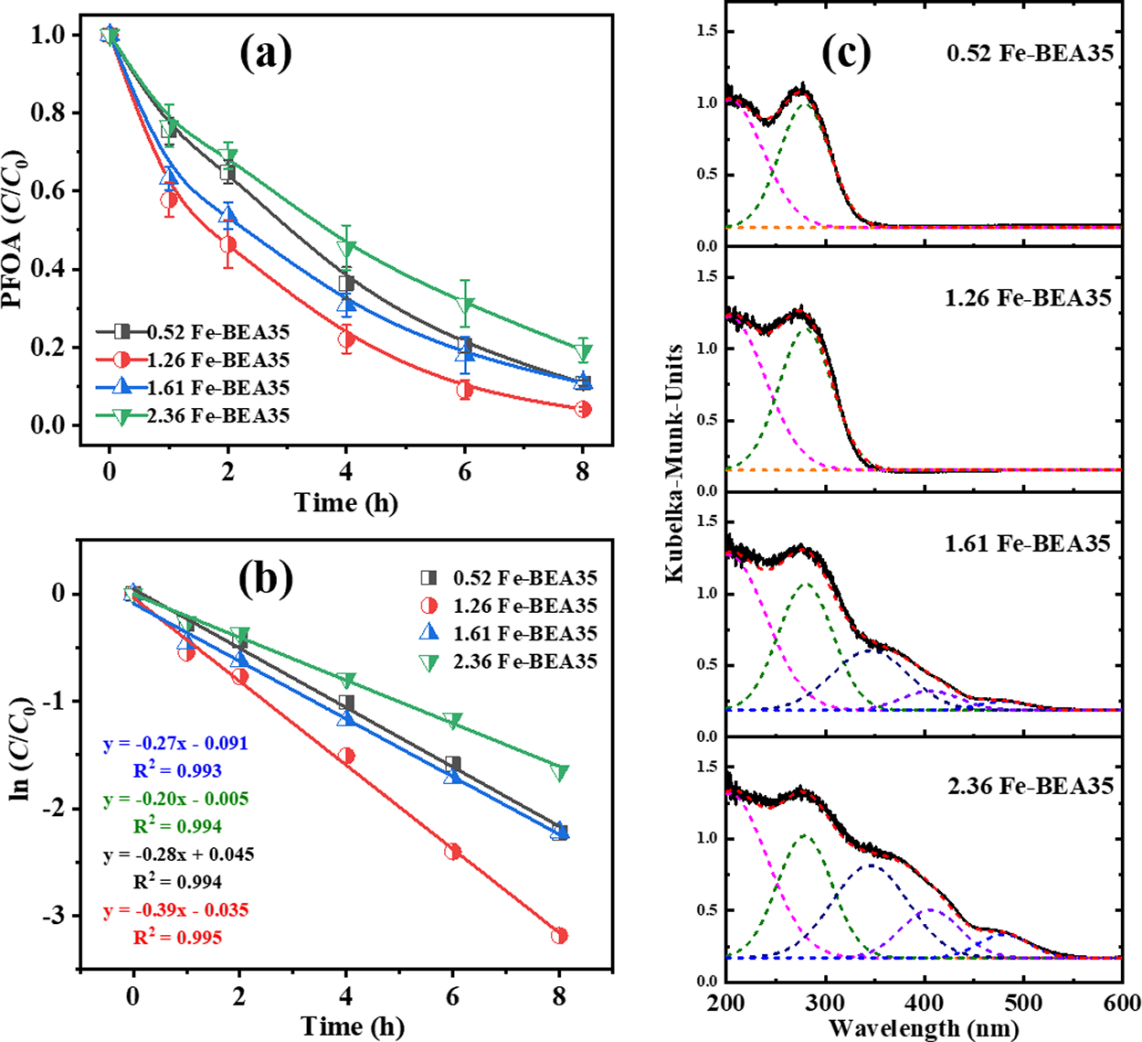
(a) Photodegradation of PFOA by Fe-BEA35 zeolites with various iron contents; (b) pseudo-first-order kinetics fit. 1 g L^–1^ zeolite, *C*_0,PFOA_ = 48 μM, pH_0_ = 5; (c) UV–vis DR spectra of iron-exchanged BEA35 zeolites with various iron contents in 10 g L^–1^ Fe-BEA35 suspension (black line). Simulated deconvolution curves for UV–vis DR spectra of the Fe-BEA35 suspension (dashed lines). Error ranges stand for the standard deviations of the results from triplicate assays in (a). Lines serve as guides for the eye in (a) and (b).

As discussed in Section 3.2, PFOA can be degraded in the Fe-zeolite under UV-A irradiation in the adsorbed state. Four ion-exchanged zeolites with various iron contents were tested for photodegradation of PFOA. As seen in Table S2, Fe-zeolites with a higher iron content show slightly smaller *X*_sorb_, but PFOA is in all cases predominantly present in the adsorbed state when photodegradation is started under the applied conditions. Ideally, a higher content of Fe^3+^ species should contribute to a higher PFOA photodegradation, as only Fe(III)-complexed PFOA can be excited and then degraded via ligand-to-metal charge transfer under UV irradiation. However, the sample 1.26 Fe-BEA35 shows the highest activity in PFOA photodegradation as well as the highest defluorination ratio within 8 h among the four Fe-BEA35 samples with iron contents from 0.52 to 2.36 wt % (Figure 3a,b). This suggests that there is an optimal iron content in the zeolite for the best PFOA photodegradation performance. This material also showed stable catalytic activity over four consecutive reuse cycles and maintained structural integrity as analyzed by XRD and XPS (Text S4, Figures S12–S14).

In order to characterize and distinguish the iron speciation in the various Fe-zeolite samples, UV–vis diffuse reflectance spectra (UV–vis DRS) were applied. As can be seen in Figure 3c, the spectra of 0.52 Fe-BEA35 and 1.26 Fe-BEA35 show strong absorption bands in the UV range (200–350 nm), while the spectra of 1.61 Fe-BEA35 and 2.36 Fe-BEA35 show broad absorption bands in the UV and visible range (200–550 nm). These absorption bands can be deconvoluted into several peaks, which have been assigned to various iron species according to the literature.^31−33^ 0.52 Fe-BEA35 and 1.26 Fe-BEA35 demonstrate absorption bands around 200 and 280 nm, which are attributed to isolated ferric ions in tetrahedral and octahedral coordination, respectively. Three more absorption bands can be identified from the spectra of 1.61 and 2.36 Fe-BEA35. The absorption band around 350 nm is assigned to octahedral ferric ions in small iron oxide clusters, and the absorption band at about 410 and 480 nm is assigned to large iron oxide particles. Several structures for isolated Fe species in Fe-loaded zeolites were hypothesized in previous papers as illustrated in Figure S7, i.e., (a) Fe species at cation exchange sites; (b) mononuclear Fe species coordinated to extra-framework Al(III); (c) framework Fe species; and (b) extra-framework Fe species tied to silicon hydroxyl nests.^34−36^

The presence of iron oxide clusters and particles in 1.61 and 2.36 Fe-BEA35 samples was also confirmed by a suspension stability test at pH 3.5. Relatively fast sedimentation of 1.61 Fe-BEA35 and 2.36 Fe-BEA35 zeolites compared with 0.52 Fe-BEA35 and 1.26 Fe-BEA35 zeolites was observed, as shown in Figure S8. Iron oxides are typically positively charged under acidic conditions (point of zero charge PZC of various iron oxides in the range of 5.5 to 9.5^37^); this leads to attractive electrostatic interactions between positively and negatively charged patches/sites on the external zeolite particle surface. Particle collisions will result in attachment and agglomeration more frequently when there is an inhomogeneous surface charge on particles. Thus, iron oxide clusters on the external surface have at least two detrimental effects: (I) they absorb/scatter light unproductively, because the majority of PFOA is adsorbed inside the zeolite pore system and oxides are not sufficiently active in PFOA degradation, as previously shown; (II) iron oxide clusters lead to agglomeration of particles, which increases shading effects and may physically block the active sites where accessible.

Nevertheless, isolated iron species bound to ion-exchange sites (tetrahedral and octahedral coordination) are reported to be more active than other iron species in redox reactions catalyzed by Fe-zeolites.^11,38^ We indeed observed the significant role of these isolated iron sites in PFOA photodegradation by relating PFOA photodegradation kinetics to iron speciation of the Fe-zeolites. The bands in UV–vis DR spectra were deconvoluted and analyzed in order to determine the different iron species semiquantitatively (Table 1), following previously published strategies.^11,31−33^ Compared to the sample with the lowest iron content, i.e., 0.52 Fe-BEA35, 1.26 Fe-BEA35 contains higher iron amounts in the form of isolated iron sites, in both tetrahedral and octahedral coordination, which contributed to a higher degradation rate of PFOA.

**Table 1:** Area Percentage of the Sub-Absorption Bands Related to Total Area (λ*n*) in UV–Vis DR Spectra from Figure 3c, Total Fe Content Determined by XRF and wt % of Fe Present in Form of the Various Iron Species (Calculated from Total Iron Content × λ*n*) for Four Fe-BEA35 Zeolites

samples	Fe-1^a^		Fe-2^b^		Fe-3^c^		Fe-4^d^		Fe-5^d^		total Fe content
	λ1 (%)	(wt %)	λ2 (%)	(wt %)	λ3 (%)	(wt %)	λ4 (%)	(wt %)	λ5 (%)	(wt %)	(wt %)
0.52 Fe-BEA35	47	0.24	53	0.28	e						0.52
1.26 Fe-BEA35	58	0.73	42	0.53							1.26
1.61 Fe-BEA35	35.3	0.57	38.9	0.63	22.9	0.37	1.5	0.02	1.4	0.02	1.61
2.36 Fe-BEA35	27.2	0.64	27.4	0.65	28.9	0.68	11.6	0.27	4.9	0.12	2.36

a Isolated Fe^3+^ ions in octahedral coordination.

b Isolated Fe^3+^ ions in tetrahedral coordination.

c Fe^3+^ ions in small iron oxide clusters.

d Large iron oxide particles.

e Not present.

Increasing the iron content further, as done for 1.61 Fe-BEA35 and 2.36 Fe-BEA35, did not further improve the photodegradation of PFOA. Although these samples show very similar amounts of isolated iron sites (1.20 and 1.29 wt %, respectively) as for 1.26 Fe-BEA35 (1.26 wt %), the latter presents the highest activity in PFOA photodegradation. It can be expected that zeolites possess a cation exchange capacity, which is limited by the number of Bro̷nsted acids and other suitable complexation sites, while also not all sites might be accessible for iron. When an excess of ferric ion is introduced during the ion-exchange processes, iron oxide clusters and particles are formed, as confirmed by the UV–vis DR spectra of 1.61 Fe-BEA35 and 2.36 Fe-BEA35 expanded to the visible range (Figure 3c). The formed iron oxide clusters and particles on the external surface of Fe-zeolite will inevitably adsorb and/or scatter UV light to some extent, such that the isolated iron sites on the internal surface of Fe-zeolite (where the majority of PFOA is adsorbed) will have less possibility to be irradiated. This then resulted in deteriorated activity in the photodegradation of PFOA.

### 3.4 Role of Internal vs External Zeolite Surface during Photodegradation of PFOA

**Figure 4:**
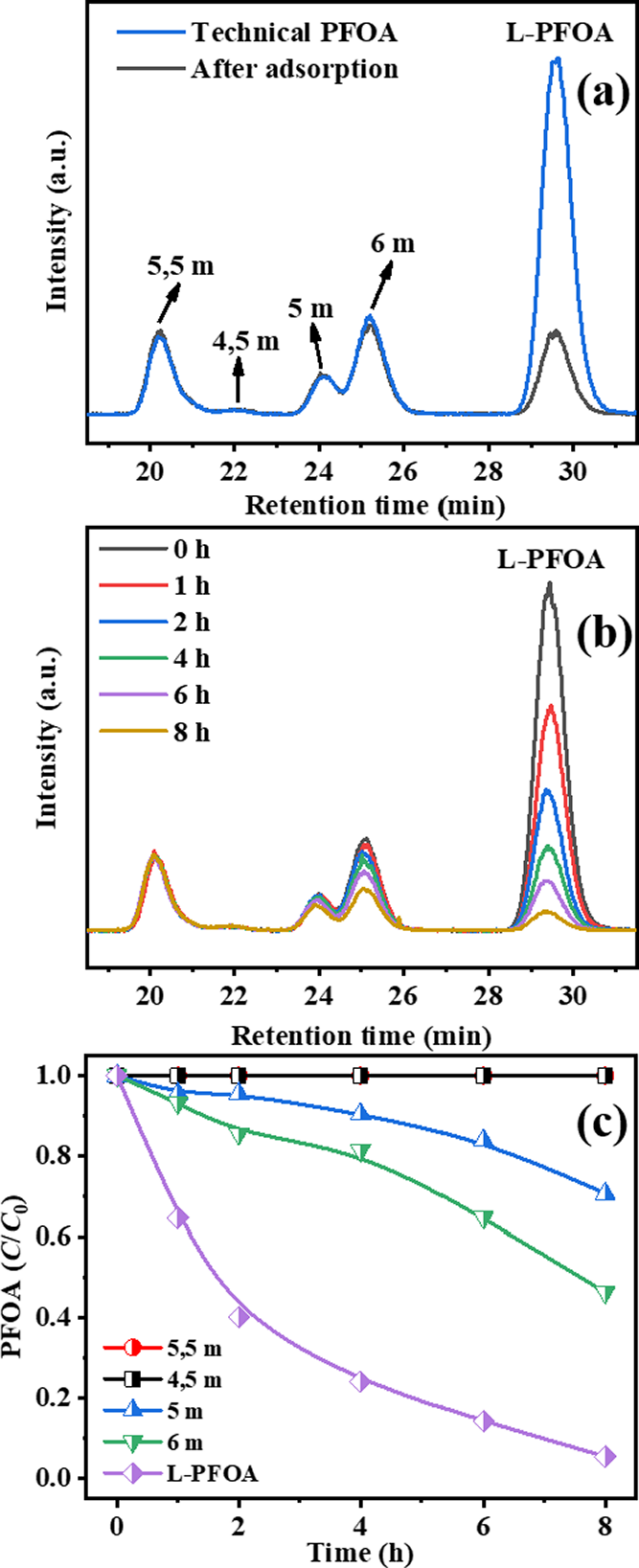
(a) LC/MS chromatograms of technical PFOA mixtures before and after 24 h of adsorption; (b) LC/MS chromatograms of technical PFOA mixtures during photodegradation; (c) kinetics of degradation of technical PFOA mixtures. Assignment of PFOA isomer standards to LC/MS chromatograms from technical PFOA. The annotations in the chromatograms represent the structure of each isomer according to the position of CF_3_-substituents in the chain, e.g., 4,5 m-PFOA = CF_3_–CF(CF_3_)–CF(CF_3_)–CF_2_–CF_2_–COOH. Lines in plots of relative concentrations are added as guides for the eye. Conditions: 1 g L^–1^ zeolite, *C*_0,PFOA_ = 48 μM, pH_0_ = 5.

BEA zeolite particles possess both internal and external surfaces. The internal surface (which consists mainly of narrow channels) can provide an appropriate environment for PFOA adsorption as discussed in Section 3.2. In addition, we illustrated in Section 3.3 that the isolated iron sites (tetrahedral and octahedral coordination) are responsible for the photodegradation of PFOA. Thus, it can be hypothesized that adsorbed PFOA is degraded mainly by the catalytic function of such isolated iron species at the internal surfaces of the zeolite. However, this requires that a significant portion of the UV irradiation penetrates the zeolite particles despite their size of 0.2 to 0.4 μm (Figure S3). In order to verify this hypothesis, a mixture of linear and branched PFOA isomers was applied as the probe (the enrichment procedures for branched PFOA isomers were described in our previous work^39^). The conception of using these PFOA isomers to explore the location of the catalytic reaction was already applied in our previous work, where the same type of zeolite (BEA35 without Fe) was shown to accelerate PFOA degradation by heat-activated persulfate.^39^ We apply this approach here for Fe-BEA35 photocatalysts for the first time. In principle, both linear and branched PFOA isomers can access the external surface, whereas the accessibility of PFOA isomers to the internal surface of the zeolite particles differs. It is reported that Fe-BEA35 has a stronger adsorption affinity toward the linear PFOA isomer than toward branched ones because the linear PFOA (effective diameter 6.0 Å^29^) can access the internal surface of Fe-BEA35 (maximum channel diameter of 6.7 Å), whereas the branched PFOA isomers can hardly access it due to size exclusion. As proved in the adsorption experiment (LC/MS chromatogram in Figure 4a, peak assignment according to PFOA isomer standards), 87% of linear PFOA can be adsorbed by Fe-BEA35, but all branched PFOA isomers can barely be adsorbed after 24 h adsorption. Subsequently, photodegradation of the PFOA isomers mixture was conducted. As seen in Figure 4b,c, linear PFOA shows the highest degradation rate among all isomers in the technical PFOA mixture. The dibranched PFOA isomers, 4,5 m-PFOA and 5,5 m-PFOA, showed negligible activity during photodegradation, as they have hardly any chance to access the narrow pores of the Fe-BEA35 zeolite due to steric effects. Surprisingly, the monobranched PFOA isomers, i.e., 6 m-PFOA and 5 m-PFOA, although showing insignificant adsorption tendency to Fe-BEA35 according to Figure 4a, were partially degraded (Figure 4b,c). Possible reasons may be that (i) the zeolite framework also has some possibilities for breathing due to vibrations, so a slow uptake for the monobranched PFOA might be plausible; (ii) the monobranched PFOA isomers can better access the internal surface of the zeolite after the adsorbed linear PFOA is degraded. The latter fits the results in Figure 4c, showing the accelerated degradation of the monobranched PFOA isomers with increasing reaction time. Overall, these results indicate that the photodegradation of PFOA takes place on the internal rather than external surface of Fe-BEA35.

### 3.5 Photodegradation of PFOA Using Various Transition-Metal-Doped BEA35 Zeolites

To date, photodegradation of PFOA has only been tested using Fe-loaded BEA35 zeolites,^11^ where a small amount of exchanged ferric ions can already cause a significant PFOA photodegradation. It is of great interest to explore whether other transition metals can be loaded into BEA35 zeolites and lead to a better photodegradation performance via ligand-to-metal charge transfer under UV-A irradiation. In addition to iron, zeolites can be ion-exchanged with a series of metal cations. Several transition metals, i.e., Mn, Cu, Co, Ni, In, and Zn, were chosen to be loaded on BEA35 zeolites and tested for their PFOA degradation efficiency under UV-A irradiation.

**Figure 5:**
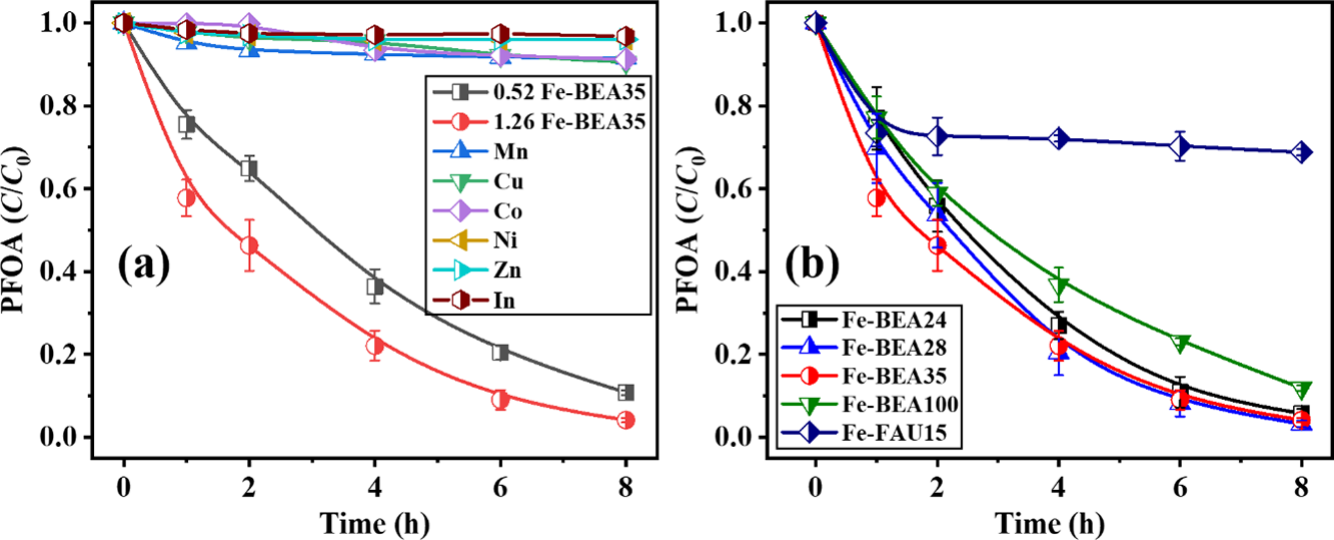
(a) Photodegradation of PFOA by transition metal ion-exchanged BEA35 zeolites; (b) photodegradation of PFOA using iron-exchanged FAU zeolite and iron-exchanged BEA zeolites with various SiO_2_/Al_2_O_3_ ratios. 1 g L^–1^ zeolites, *C*_0,PFOA_ = 48 μM, pH_0_ = 5. Error ranges stand for the standard deviations of the results from triplicate assays. Lines serve as guides for the eye.

After a preadsorption period of 1 day, PFOA was in all cases mainly present in the adsorbed state (80–90%) in the 1 g L^–1^ suspensions of the transition-metal-loaded BEA35 zeolites. The UV irradiation was conducted afterward. According to Table S3, different ion-uptake efficiencies of the transition metal ions were observed, ranging from 0.7 to 27% with the same initial dosage (1 mM of each metal salt), which is probably due to the different hydrated metal ion radii affecting zeolite exchange capacities. As can be seen in Figure 5a, all transition metal ion-exchanged zeolites, except those with Fe, show negligible activities on PFOA photodegradation, regardless of the various ion-uptake efficiencies and metal to Al molar ratios, presumably for one or more of the following reasons in each case: (a) the metal uptake by the zeolite is too low (as in the case of Zn and In); (b) the complex between transition metal ions and PFOA is not formed; (c) the complex is formed but cannot be excited and decomposed under UV-A irradiation, i.e., the ligand-to-metal charge transfer cannot take place.

### 3.6 Photodegradation of PFOA Using Other Types of Zeolites

Since no significant enhancement was found when loading BEA35 zeolites with other transition metal ions, we investigated whether a change in the zeolite type can lead to any improvement. In addition to Fe-BEA35, other BEA-type zeolites with different molar SiO_2_/Al_2_O_3_ ratios, i.e., BEA24, BEA28, BEA100, and a zeolite of the faujasite type, i.e., FAU15, were chosen to be loaded with iron and tested for PFOA photodegradation. For all zeolites, the same conditions, i.e., 10 g zeolite in 1 mM iron(II) sulfate heptahydrate solution were applied for ion exchange.

As discussed in Section 3.3, when the same BEA-type zeolite is in use (i.e., the same SiO_2_/Al_2_O_3_ ratio), a higher amount of isolated iron sites should contribute to a higher photodegradation rate *k*_obs_. Theoretically, decreasing the SiO_2_/Al_2_O_3_ ratio leads to a higher cation exchange capacity (higher amount of isolated iron sites) as the number of AlO_4_^–^ sites is increasing. On the contrary, however, PFOA adsorption benefits when the SiO_2_/Al_2_O_3_ ratio is increased due to a higher surface hydrophobicity.^40^ In addition, for the Fe-exchanged zeolites, a larger fraction of adsorbed PFOA (*X*_sorb_, Table S4) is observed with an increasing SiO_2_/Al_2_O_3_ ratio. As shown in Figure 5b and Table S4, we observed a similarly high PFOA degradation efficiency of Fe-BEA24, Fe-BEA28, and Fe-BEA35 despite the large difference in *X*_sorb_, whereas a significantly lower PFOA degradation efficiency of Fe-BEA100 can be seen. Apparently, the two counteracting effects—improved PFOA adsorption vs fewer ion-exchange sites with increasing SiO_2_/Al_2_O_3_ ratio—cancel each other out within moderate changes of SiO_2_/Al_2_O_3_ until a too low Al content eventually severely limits the binding capacity for active iron and thus catalytic activity. Fe-BEA35 shows comparable and the best PFOA adsorption performance, which is needed for the desired preconcentrate-and-degrade approach. Thus, its adsorption performance was investigated in more detail in Text S2 in the SI. PFOA adsorption can be well fitted by the Freundlich isotherm in the range of aqueous phase PFOA concentrations of 0.7 to 700 μg L^–1^, with a Freundlich coefficient of *K*_F_ = 10^4.5^ mg^1−*n*^ kg^–1^ L^−*n*^ and *n* = 0.63 and the highest *K*_d_ determined at the lowest tested *C*_free_ = 0.7 μg L^–1^ is 6.0 × 10^5^ L kg^–1^.

We then tested Fe-FAU15 for PFOA photodegradation. Although 93% of PFOA (*X*_sorb_ = 0.93) was in the adsorbed state before starting the irradiation, only 20% of PFOA was degraded at the beginning (within 1 h), after which the reaction was terminated. Note that in the high PFOA loading range studied here (about 2 wt %), PFOA sorption to FAU is high (similar to Fe-BEA100). The much poorer PFOA photodegradation efficiency despite large *X*_sorb_ implies a different iron speciation in FAU15 zeolite with its cage-dominated pore volume. In FAU supercages, catalytically active isolated iron sites could be either limited or separated from the sites for PFOA adsorption. In the other scenario, the distance between the isolated iron sites in the zeolite framework and the PFOA in the central volume of the filled pore may be too large for complex formation. In contrast, the close fit of chainlike PFOA molecules in the straight channels of the BEA zeolite and its ability to stabilize isolated iron species are more favorable for the catalytic photodegradation.

## 4 Conclusions

In this study, we have investigated the metal ion-exchanged zeolites as catalysts for the photodegradation of PFOA. Experimental results show that (i) the isolated iron species (in tetrahedral and octahedral coordination) are active in photodegradation of PFOA, while iron oxide clusters and larger particles hinder the photodegradation of PFOA; (ii) photodegradation of PFOA takes place on the internal surface rather than the external surface of Fe-BEA35 particles; (iii) the iron-exchanged beta type zeolite (Fe-BEA) can be applied for photodegradation of PFOA, while other transition metal (i.e., Mn, Cu, Co, Ni, In, and Zn) ion-exchanged BEA zeolites show negligible photochemical activities in the degradation of PFOA; (iv) iron-exchanged BEA-type zeolites with various SiO_2_/Al_2_O_3_ ratios show sufficient activities for photodegradation of PFOA, while iron-exchanged FAU-type zeolites exhibit negligible activities; and (v) BEA zeolites with moderate SiO_2_/Al_2_O_3_ ratio of about 30 show the best compromise between PFOA adsorption performance and catalytic activity. Overall, we successfully optimized the transition metal ion-exchanged zeolites for effective photodegradation of PFOA and on-site regeneration in the scale of this study. This research paved the way for next-generation zeolite-based photocatalyst design and will contribute to process development for the treatment of PFAS and other recalcitrant trace-level contaminants through combined pre-enrichment and degradation approaches. However, this requires parallel research into reactor design and particle separation strategies for suspended particle applications. Fixed-bed Fe-zeolite photocatalyst applications may be even more preferable due to their easier scale-up and operation.

## Supporting Information Available

Detailed description of experimental procedures, characterization parameters for Fe-BEA35, comparison of PFOA adsorption by various zeolite framework types, isotherms for PFOA adsorption on BEA35, results of stability and reusability tests, and mechanistic scheme explaining different PFOA sorption sites (PDF)
