# Supplementary material for: A Deep Insight into Perfluorooctanoic Acid Photodegradation Using Metal Ion-Exchanged Zeolites
Source: ACS ES T Eng. 2024 Feb 15;4(3):748–57. doi: 10.1021/acsestengg.3c00462 (PMC10928708; doi:10.1021/acsestengg.3c00462)
Supplement: Supplementary file 1 — ee3c00462_si_001.pdf [file ee3c00462_si_001.pdf]

Supporting information

A deep insight into perfluorooctanoic acid photodegradation using metal ion-exchanged zeolites

Lin Qian[a,b], Hongying Zhao[b], Ariette Schierz[a], Katrin Mackenzie[a] and Anett Georgi[a]*

[a] Helmholtz Centre for Environmental Research – UFZ, Department of Environmental Engineering, Permoserstr. 15, D-04318, Leipzig, Germany

[b] School of Chemical Science and Engineering, Shanghai Key Lab of Chemical Assessment and Sustainability, Key Laboratory of Yangtze River Water Environment, Tongji University, 1239 Siping Road, Shanghai 200092, China

Corresponding author e-mail: anett.georgi@ufz.de

Contents

Text S1: Detailed information on Experimental Section ... 4

Chemicals and materials. ... 4

Catalyst preparation. ... 4

Photochemical degradation. ... 5

Analysis. ... 5

Text S2: PFOA adsorption to BEA35 zeolites ... 7

Text S3: Photodegradation of PFOA under UV irradiation in the presence of ferric ions and Fe-zeolites. . 8

Text S4: Stability and reusability test ... 9

Tables S1-S5 referred to in the main text and supporting information ... 11

Table S1. Selected characterization parameters of iron-exchanged BEA35 zeolites ... 11

Table S2. Adsorption and kinetic data of PFOA degradation by Fe-BEA35 zeolites with various iron contents. Reaction conditions: 1 g L^{-1} zeolite, $C_{0,\mathrm{PFOA}}$ = 48 μM, pH_0 = 5, 1 day adsorption equilibrium before irradiation ... 11

Table S3. Selected parameters of transition metal ion-exchanged BEA35 zeolites. Several metal ion solutions with 1 mM concentration were used for ion-exchange into zeolites during the preparation (for details see section 2.2). ... 12

Table S4. Adsorption and kinetic data on PFOA degradation using iron-exchanged FAU zeolite and iron-exchanged BEA zeolites with various SiO_2/Al_2O_3 ratios. Reaction conditions: 1 g L^{-1} zeolite, $C_{0,\mathrm{PFOA}}$ = 48 μM, pH_0 = 5, 1 day pre-adsorption before irradiation. ... 13

Table S5. List of tested zeolites with additional information. ... 13

Figures S1 to S12, referred to in the main text and supporting information. ... 14

Figure S1. Overview of logarithmized single-point adsorption coefficients K_d of zeolites with various framework structures (purple bars: BEA type, green bars: FAU type, orange bars: MFI type, and yellow bar: activated carbon) and various SiO_2/Al_2O_3 molar ratios (value behind framework code) at pH 7 in 10 mM KNO_3 with four days' adjustment of adsorption equilibrium using 5.5 mg L^{-1} PFOA and 2 g L^{-1} zeolite, resulting in $C_{\mathrm{free,PFOA}}$ = 10 to 20 µg L^{-1} for BEA zeolites. ... 14

Figure S2. Scheme of the experimental setup for photochemical experiments. ... 15

Figure S3. Scanning electron microscope (SEM) images of iron-exchanged BEA35 zeolites with various iron contents: (a) BEA35, (c) 0.52 Fe-BEA35, (d) 1.26 Fe-BEA35, (e) 1.61 Fe-BEA35, and (f) 2.36 Fe-BEA35; (b) the particle size distribution of BEA35 zeolites. ... 16

Figure S4. Distribution of PFCAs with various chain lengths between zeolite 1.26 Fe-BEA35 (X_{sorb}) and the aqueous phase (X_{free}) after 1 day of shaking. 1 g L^{-1} 1.26 Fe-BEA35, $C_{0,\mathrm{PFCAs}}$ = 48 μM and pH_0 = 5. PFCAs with chains of 2 and 3 carbons did not show a significant sorption effect. ... 17

Figure S5. Schematic diagram of PFOA configurations on Fe-BEA35 with and without specific adsorption. The term "complexed PFOA" means the specifically adsorbed PFOA at ferric ions. ... 18

Figure S6. Photodegradation of PFOA by Fe-BEA35 zeolite at various initial pH values. 1 g L^{-1} 1.26 Fe-BEA35, $C_{0,\mathrm{PFOA}}$ = 48 μM. Error ranges stand for the standard deviations of the results from triplicate assays. Lines serve as guides for the eye. ... 19

Figure S7. Schematic diagram of four isolated Fe species (as examples) in Fe-containing zeolites catalysts. (a) Fe species at cation exchange sites; (b) mono-nuclear Fe species coordinated to extra-

framework Al(III); (c) framework Fe species; and (b) extra-framework Fe species tied to silicon hydroxyl nests. [12-14] 20

Figure S8. Stability of aqueous zeolite suspensions (2.5 g L^{-1} zeolite in 100 mM KNO_3, pH=3.5) determined by sedimentation analysis (continuous time-resolved measurement of relative absorbance A/A_0 at 725 nm at a depth of 20 mm below water level). 21

Figure S9. Spectral beam intensity of UV-A lamp. The intensity varies along with power of the lamp. The spectral curve was acquired from the UV lamp provider (https://www.herolab.de). 22

Figure S10. Adsorption isotherms (a) and single-point sorption coefficients as function of equilibrium aqueous phase PFOA concentration (b) for adsorption of PFOA on BEA35 zeolite in EPA standard soft water, pH 7.[5] 23

Figure S11. Adsorption isotherm for adsorption of PFOA on BEA35 zeolite in EPA standard soft water, pH = 7 fitted by the Langmuir equation (for data points with $q_m \geqslant 5$ g kg^{-1} or 0.5 wt.%) 24

Figure S12. Fe 2p XPS spectra of 1.26 Fe-BEA35 before and after photodegradation of PFOA. 25

Figure S13. The X-ray powder diffraction pattern of 1.26 Fe-BEA35 before and after photodegradation of PFOA 26

Figure S14. Reusability test of Fe-zeolite for PFOA degradation under UV-A irradiation. 1 g L^{-1} 1.26 Fe-BEA35, $C_{0,PFOA}$ = 48 μM each, pH_0 = 5. Lines serve as guides for the eye. 27

Figure S15: Kinetics of PFOA adsorption on Fe-BEA35 zeolite (10 g L^{-1} 1.26 Fe-BEA35, $C_{0,PFOA}$ = 48 μM) 28

References. 29

Text S1: Detailed information on Experimental Section

Chemicals and materials.

All chemicals were of reagent grade and used as received. Deionized (DI) water was utilized for preparation of solutions and suspensions. Perfluorooctanoic acid (PFOA, $C_7F_{15}COOH$, 96 %), perfluorobutanoic acid (PFBA, C_3F_7COOH, 98 %), hydrogen peroxide solution (30 % w/w in H_2O) and iron(III) oxide powder (< 50 nm particle size) were obtained from Sigma Aldrich. Perfluoroheptanoic acid (PFHpA, $C_6F_{13}COOH$, 97 %) and zinc sulfate heptahydrate were obtained from Alfa Aesar. Perfluorohexanoic acid (PFHeA, $C_5F_{11}COOH$, 98 %), perfluoropropanonic acid (PFPrA, C_2F_5COOH, 97 %) and perfluoropentanoic acid (PFPeA, C_4F_9COOH, 97 %) were obtained from J&K Scientific. Trifluoroacetic acid (TFA, CF_3COOH, 99 %) and copper(II) sulfate pentahydrate were obtained from Fluka. The technical PFOA, containing branched PFOA isomers, was obtained from Haihang Industry (China). Indium(III) chloride was obtained from Carl Roth. Iron(II) sulfate heptahydrate, cobalt(II)-nitrate hexahydrate, nickel(II) sulfate hexahydrate, and manganese(II) sulfate monohydrate were obtained from Merck. The BEA24 and BEA35 zeolites were obtained from Clariant Produkte GmbH, Germany. The FAU15, BEA28 and BEA100 were obtained from Tosoh Corporation. More detailed information about the tested zeolites can be found in Table S5.

Catalyst preparation.

In this study, commercially available BEA type and FAU type zeolites were used for preparing metal cation-exchanged zeolites. The procedure is based on our previous study and described as follows [1]: (a) 10 g of target zeolite is suspended in 100 mL DI water and treated in an ultrasonic bath for 15 min; (b) after stirring with nitrogen purging for 30 min, a certain amount of target metal salts is added under nitrogen atmosphere; (c) the pH of the zeolite suspension is adjusted to 3.0 with H_2SO_4 or NaOH under nitrogen atmosphere; (d) the vial is then closed and heated at 90 °C with stirring overnight; (e) the zeolite sample is separated from the liquid phase by centrifugation and washed with DI water three times; (f) the zeolite sample is then re-

suspended in 100 mL 10 wt. % H_2O_2 solution and stirred overnight; (g) the zeolite sample is separated from the liquid phase by centrifugation, washed with DI water three times, and finally dried at 105 °C overnight.

Photochemical degradation.

The reaction setup has been described in our previous work.[2] Where not otherwise stated, 30 mL aqueous PFOA solution (48 μM) was mixed with 0.03 g of metal-cation-exchanged zeolite in a 40 mL quartz reactor followed by 24 h shaking in order to approach adsorption equilibrium. The reactor was shaken on a rotating shaker with 240 rpm constantly during the photochemical process in order to guarantee a good dispersion of the zeolite in the suspension. The scheme of the photochemical setup is shown in Figure S2. The UV-A mercury lamp (6 W, central wavelength 365 nm, Herolab GmbH Laborgeräte, Germany) was placed beneath the quartz reactor. The distance between the bottom of the quartz reactor and the UV lamp window was 20 mm. The spectral curve of the UV-A lamp is shown in Figure S9. The photon flux was measured by ferrioxalate actinometry to be 4.47×10^{-6} mol s^{-1}.

Analysis.

In order to determine the total concentrations (freely dissolved and adsorbed fraction) of PFOA and shorter-chain acids (C4 - C7) in the zeolite suspension, 0.1 mL aliquots of the suspension were transferred into a 4 mL vial and the pH was adjusted to below 2. Afterwards, 2 mL acetonitrile was added and shaken for 24 h to extract the adsorbed fractions into the liquid phase. The suspension was separated by centrifugation and the clear supernatant was transferred to LC/MS analysis. In the current study, the PFOA recovery after this extraction process was 94 ± 3 %. In order to determine the concentrations of fluoride and shorter-chain acids (C2 and C3) in the suspension, 1 mL aliquots were taken and the pH was adjusted to ≥ 11. The suspension was shaken for 2 h, separated by centrifugation and the clear supernatant was used for IC analysis.

The concentration of PFOA and shorter-chain acids (C4-C7) were determined by LC/MS (LCMS-2020; SHIMADZU Corp.), and the concentration of fluoride and shorter-chain acids (C2-C3) were determined by ion chromatography (IC, Dionex) as described.[3, 4] The defluorination ratio (d_{F^-}) is calculated as follows:

$$d_{F^-} = \frac{C_{F^-}}{15 \times C_0} \times 100\ \% \qquad (1)$$

where C_{F^-} is the fluoride concentration (μM) and C_0 is the initial concentration of PFOA (μM). The factor 15 represents the number of fluorine atoms in one PFOA molecule.

UV–vis diffuse reflectance spectroscopy was performed in a Varian Cary 3 spectrophotometer featuring a range of 190 to 600 nm. 10 g L^{-1} zeolite suspension was filled into a 0.5 cm cuvette and placed into the sample holder where powdered BaSO4 was applied as the non-absorbing reference standard. The spectra were converted into equivalent absorption spectra by applying the Kubelka-Munk function and the bands were deconvoluted into sub-bands with Gaussian peak shape using OriginLab 2018.

Major elements of the zeolites were determined by means of wavelength-dispersive X-ray fluorescence analysis (WDXRF) using a WDXRF-spectrometer S4 PIONEER (Bruker-AXS) equipped with a 4 kW-Rh X-ray tube (75 µm Be window), 60 kV generator and an eight-position crystal changer. Aliquots of the sample powder were diluted with silicic acid (1 g sample + 7 g silicic acid) in order to prepare glass discs by fusion at 1200 °C in a 95 % platinum / 5 % gold crucible for 20 min. The calibration curves for the individual analytes were generated based on the relationship between the certified concentration data and the measured responses of the reference materials: CANMET-LKSD1-LKSD4 (lake sediments), CANMET-STSD1-STSD4 and GBW07309-11 (stream sediments), NIST-SRM2689 and NIST-SRM2691 (coal fly ashes).

XPS spectra were recorded on an Axis Ultra photoelectron spectrometer (Kratos, Manchester, UK) using monochromatized Al Kα radiation (hν=1486.6 eV). Pass energies of 160 eV and 40 eV were set for measuring the survey and the high-resolution spectra, respectively. The main component of the C 1s signal was set as reference at 284.8 eV for binding energy determination.

X-ray powder diffraction patterns were recorded at room temperature on a Bruker D8-Advance diffractometer, equipped with a one-dimensional silicon strip detector (LynxEye) using Cu-K radiation and a counting time of 1 s per data point.

Text S2: PFOA adsorption to BEA35 zeolites.

So far, Fe-loaded BEA zeolites with moderate SiO_2/Al_2O_3 in the range of about 30 are recognized as suitable photodegradation catalysts. With a half-life ($\tau_{½}$) in the range of 1.6 to 1.8 h, PFOA photodegradation is, however, relatively slow. Thus, the reaction cannot realistically be applied to treat large volumes of PFOA-contaminated water directly. Nevertheless, the Fe-BEA zeolites differ from other photocatalysts in their sorption performance for PFOA, which allows a concentrate-and-degrade strategy to be applied. This means that the PFOA is firstly separated from the contaminated water by fast and safe adsorption to the Fe-BEA zeolite, and afterwards degraded in the adsorbed state using UV-irradiation, which in turn regenerates the adsorbent. In order to characterize in more detail the sorption properties of BEA35 which showed the best compromise between adsorption and catalytic activity, adsorption isotherms were obtained in standard soft water [5] for PFOA.

As Figure S10(a) shows, PFOA adsorption can be well fitted by the Freundlich isotherm in the range of aqueous phase PFOA concentrations of 0.7 to 700 µg L^{-1}, with a Freundlich coefficient of $K_F = 10^{4.5}$ mg^{1-n} kg^{-1} L^{-n} and $n = 0.63$. Beyond this range, sorption is approaching maximum loading, which was determined by fitting the data in the higher concentration range to the linearized Langmuir isotherm with $q_{max} = 7.7$ wt. % (Figure S11).

Compared to an all-silica BEA zeolite, the maximum loading of the BEA zeolite with moderate Al content (and thus uptake capacity for Fe) is significantly lower (7.8 vs. 35 wt. %). This maximum loading is relevant for PFOA removal from high-concentrated solutions in the tens of mg L^{-1} range. However, PFOA contamination in the environment is frequently detected in the ng to tens of µg L^{-1} concentration range. Due

to the non-linear isotherm shape, K_d values increase with decreasing PFOA aqueous phase concentration (Figure S10(b)), which is beneficial for adsorptive enrichment. The highest K_d determined at C_{free} = 0.7 µg L^{-1} in this study for the BEA35 zeolite is 6.0×10^5 L kg^{-1}, while at C_{free} = 10 µg L^{-1} it is still 1.5×10^5 L kg.[6] This ranks the BEA35 zeolite among the very good adsorbents for PFOA. While some organic and/or carbon-based adsorbents (ion exchange resins, cyclodextrin polymers or high-performance activated carbons as summarized by Aumeier et al.[7]) show even higher K_d values up to 10^6 L kg^{-1} at C_{free} in the µg L^{-1} range, the Fe-loaded zeolite has the advantage of regeneration simply by sunlight.

Text S3: Photodegradation of PFOA under UV irradiation in the presence of ferric ions and Fe-zeolites.

The aquatic ferric ions mediated PFOA photodegradation mechanism were reported in the literature.[8-11] The PFOA photodegradation by Fe-exchanged BEA (Fe-BEA) zeolites is similar to the ferric ions mediated approach except the initial steps (eqs.1-2).

In the first step, Fe-BEA will adsorb PFOA in water, and some fraction of PFOA present as complexed PFOA ($[C_7F_{15}COO\text{-}Fe]^{2+}$-zeolite, eq. S1). The PFOA complex can be excited under UV-A irradiation to produce Fe^{2+}-zeolite and $C_7F_{15}COO\cdot$ via carboxyl-to-metal electron transfer (eq. S2).

$$C_7F_{15}COO^- + Fe^{3+}\text{-zeolite} \longleftrightarrow [C_7F_{15}COO\text{-}Fe]^{2+}\text{-zeolite} \qquad \text{(S1)}$$

$$[C_7F_{15}COO\text{-}Fe]^{2+}\text{-zeolite} \xrightarrow{\text{UV-A}} C_7F_{15}COO\cdot + Fe^{2+}\text{-zeolite} \qquad \text{(S2)}$$

The produced $C_7F_{15}COO\cdot$ is not stable, and will decarboxylate afterward to produce perfluoroalky radical ($C_7F_{15}\cdot$) and CO_2 (eq. S3).

$$C_7F_{15}COO\cdot \rightarrow C_7F_{15}\cdot + CO_2 \qquad \text{(S3)}$$

The C_7F_{15} will preferentially react with dissolved oxygen to form $C_7F_{15}OO$ (eq. S4).

$C_7F_{15}\cdot + O_2 \rightarrow C_7F_{15}OO\cdot$ (S4)

The subsequent radical and hydrolysis reactions have been proposed in the literature (eqs. S5 - S9).

$2C_7F_{15}OO\cdot \rightarrow C_7F_{15}OOOOC_7F_{15}$ (S5)

$C_7F_{15}OOOOC_7F_{15} \rightarrow 2C_7F_{15}O\cdot + O_2$ (S6)

$C_7F_{15}O\cdot + H_2O \rightarrow C_7F_{15}OH + \cdot OH$ (S7)

$C_7F_{15}OH \rightarrow C_7F_{13}OF + HF$ (S8)

$C_7F_{13}OF + H_2O \rightarrow C_6F_{13}COOH + HF$ (S9)

The produced $C_6F_{13}COOH$ with one CF_2 unit less than PFOA can complex again with ferric ions and be decomposed further until complete mineralization. Additionally, the produced Fe^{2+}-zeolite can be re-oxidized by molecular oxygen into Fe^{3+}-zeolite, and the photocatalytic cycle is formed (eq.S10).

$Fe^{2+}\text{-zeolite} + O_2 \rightarrow Fe^{3+}\text{-zeolite} + \cdot O_2^-$ (S10)

Text S4: Stability and reusability test

The surface chemical states of introduced Fe in the Fe-BEA35 before and after reaction was analyzed by X-ray photoelectron spectroscopy (XPS). The broad Fe 2p spectra were deconvoluted into several peaks and fitted according to the literature (Figure S12) [15,16]. The peak at 710.4 eV was assigned to Fe(II) species. The peaks at 712.0 and 714.1 eV were assigned to Fe(III) species, which may be ascribed to the ferric ions in octahedral and tetrahedral coordination, respectively. These XPS results fit well with our expectations that Fe-BEA35 consists mainly Fe(III) species, which contain active sites for PFOA photodegradation. The fraction of Fe(II) species slightly increased after reaction (from 19% to 23%), yet the major fraction of Fe(III) species was not affected (from 81% to 77%). To evaluated the stabilities of crystallographic structure

of Fe-BEA35 before and after photodegradation of PFOA, the X-ray powder diffraction (XRD) measurement was performed (Figure S13). As seen in Figure x, almost identical XRD patterns were obtained for the Fe-BEA35 before and after reaction, which indicated the relatively stable crystallographic structure of Fe-BEA35.

In view of practical applications, experiments on the reusability of 1.26 Fe-BEA35 for photodegradation of PFOA were performed (Figure S12). After each 8 h irradiation period, the supernatant was separated via centrifugation and replaced by fresh aqueous PFOA solution with the same volume. The zeolite suspension was prepared for the next reaction run after 1 day-shaking for adsorption equilibration. As seen in Figure S12, the 1.26 Fe-BEA35 continues to exhibit a relatively high activity for PFOA photodegradation after four consecutive runs, as only a slight decrease in PFOA degradation performance was observed. The possible reasons for this decrease might be: (i) the remaining PFOA and its byproducts (PFCAs) remain on the zeolite between the runs; (ii) some zeolite separation losses during reusability tests; (iii) the slightly decreased fraction of Fe(III) species relative to Fe(II) after the catalytic reaction compared to the fresh material. As the inherent catalyst activity is not affected during the reusability test, it shows good stability and is thus suitable potential for practical application.

Tables S1-S5 referred to in the main text and supporting information

Table S1. Selected characterization parameters of iron-exchanged BEA35 zeolites.

Sample	Dosed Fe ($g\ kg^{-1}$)	Detected Fe ($g\ kg^{-1}$)	Ion-uptake efficiency (%)	Fe content (wt. %)	Fe/Al (mol/mol)
0.52 Fe-BEA35	14	5.2	37	0.52	0.10
1.26 Fe-BEA35	56	12.6	22	1.26	0.25
1.61 Fe-BEA35	113	16.1	14	1.61	0.32
2.36 Fe-BEA35	226	23.6	10	2.36	0.48

Table S2. Adsorption and kinetic data of PFOA degradation by Fe-BEA35 zeolites with various iron contents. Reaction conditions: 1 g L^{-1} zeolite, $C_{0,PFOA}$ = 48 µM, pH_0 = 5, 1 day adsorption equilibrium before irradiation.

Sample	X_{sorb}	q_{PFOA} ($g\ kg^{-1}$) [a]	K_d ($L\ kg^{-1}$) [b]	k_{obs} (h^{-1})	$\tau_{½}$(h)
0.52 Fe-BEA35	0.86	17.3	6.4×10^3	0.28 ± 0.018	2.51 ± 0.16
1.26 Fe-BEA35	0.83	16.5	4.7×10^3	0.39 ± 0.024	1.77 ± 0.10
1.61 Fe-BEA35	0.82	16.4	4.6×10^3	0.27 ± 0.020	2.58 ± 0.18
2.36 Fe-BEA35	0.82	16.5	4.6×10^3	0.20 ± 0.014	3.46 ± 0.22

[a] Loading of PFOA on zeolite.

[b] Single-point adsorption coefficient K_d ($L\ kg^{-1}$) = q_{PFOA} ($g\ kg^{-1}$)/$C_{PFOA,free}$ ($g\ L^{-1}$)

Table S3. Selected parameters of transition metal ion-exchanged BEA35 zeolites. Several metal ion solutions with 1 mM concentration were used for ion-exchange into zeolites during the preparation (for details see section 2.2).

Sample	Added metal (g kg^{-1})	Detected metal (g kg^{-1})	Ion-uptake efficiency (%)	Metal content (%)
0.52 Fe-BEA35	14	5.2	37	0.52
1.26 Fe-BEA35	56	12.6	22	1.26
Co-BEA35	59	5.5	9	0.56
Ni-BEA35	59	3.2	5	0.32
Cu-BEA35	64	3.4	5	0.34
Zn-BEA35	65	0.4	0.7	0.04
Mn-BEA35	55	14.8	27	1.48
In-BEA35	115	0.23	0.2	0.02

Table S4. Adsorption and kinetic data on PFOA degradation using iron-exchanged FAU zeolite and iron-exchanged BEA zeolites with various SiO_2/Al_2O_3 ratios. Reaction conditions: 1 g L^{-1} zeolite, $C_{0,PFOA}$ = 48 μM, pH_0 = 5, 1 day pre-adsorption before irradiation.

**Sample**	X_{sorb}	q_{PFOA} **(g kg^{-1})**	K_d **(L kg^{-1})**	k_{obs} **(h^{-1})**	$\tau_{½}$ **(h)**
Fe-BEA24	0.45	9.02	8.2×10^2	0.44 ± 0.048	1.56 ± 0.27
Fe-BEA28	0.68	13.64	2.1×10^3	0.43 ± 0.014	1.59 ± 0.09
Fe-BEA35	0.83	16.51	4.7×10^3	0.39 ± 0.012	1.77 ± 0.10
Fe-BEA100	0.95	19.06	2.0×10^4	0.26 ± 0.008	2.67 ± 0.15
Fe-FAU15	0.93	18.67	1.4×10^4	-[a]	-[a]

[a] Negligible PFOA degradation observed within 8 h.

Table S5. List of tested zeolites with additional information.

**Zeolite[a]**	**Supplier**	**Supplier's designation**	SiO_2/Al_2O_3 **(mol/mol)[b]**	**BET surface area (m^2 g^{-1})[c]**
BEA24	Clariant	TZB213	24	660
BEA28	Tosoh	931HOA	28	510
BEA35	Clariant	TZB223L	35	602
BEA100	Tosoh	960HOA	100	560
FAU15	Tosoh	360HUA	15	550

[a] Zeolite designation: three-letter code (presents framework type code given by International Zeolite Association), and the following number represents the SiO_2/Al_2O_3 molar ratio.

[b] The SiO_2/Al_2O_3 molar ratio was provided by the supplier.

[c] The BET surface area was provided by supplier or determined by N_2 adsorption/desorption experiment.

Figures S1 to S15, referred to in the main text and supporting information.

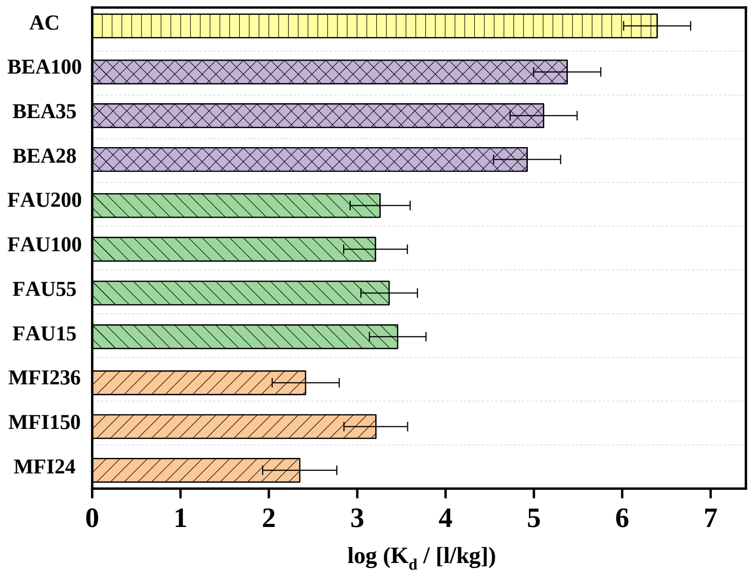

Figure S1. Overview of logarithmized single-point adsorption coefficients K_d of zeolites with various framework structures (purple bars: BEA type, green bars: FAU type, orange bars: MFI type, and yellow bar: activated carbon) and various SiO_2/Al_2O_3 molar ratios (value behind framework code) at pH 7 in 10 mM KNO_3 with four days' adjustment of adsorption equilibrium using 5.5 mg L^{-1} PFOA and 2 g L^{-1} zeolite, resulting in $C_{free,PFOA}$ = 10 to 20 µg L^{-1} for BEA zeolites.

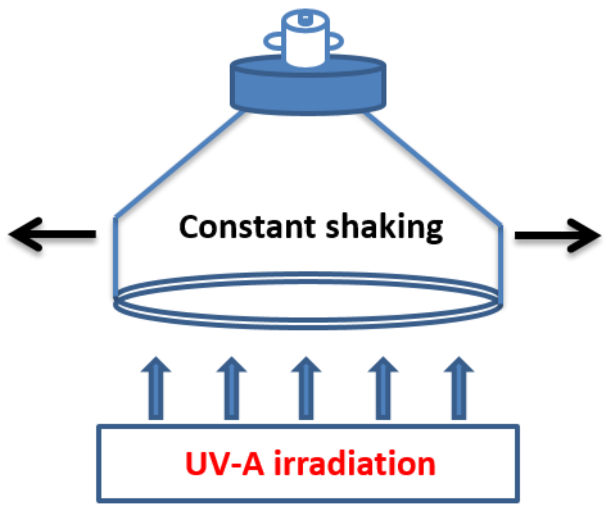

Figure S2. Scheme of the experimental setup for photochemical experiments.

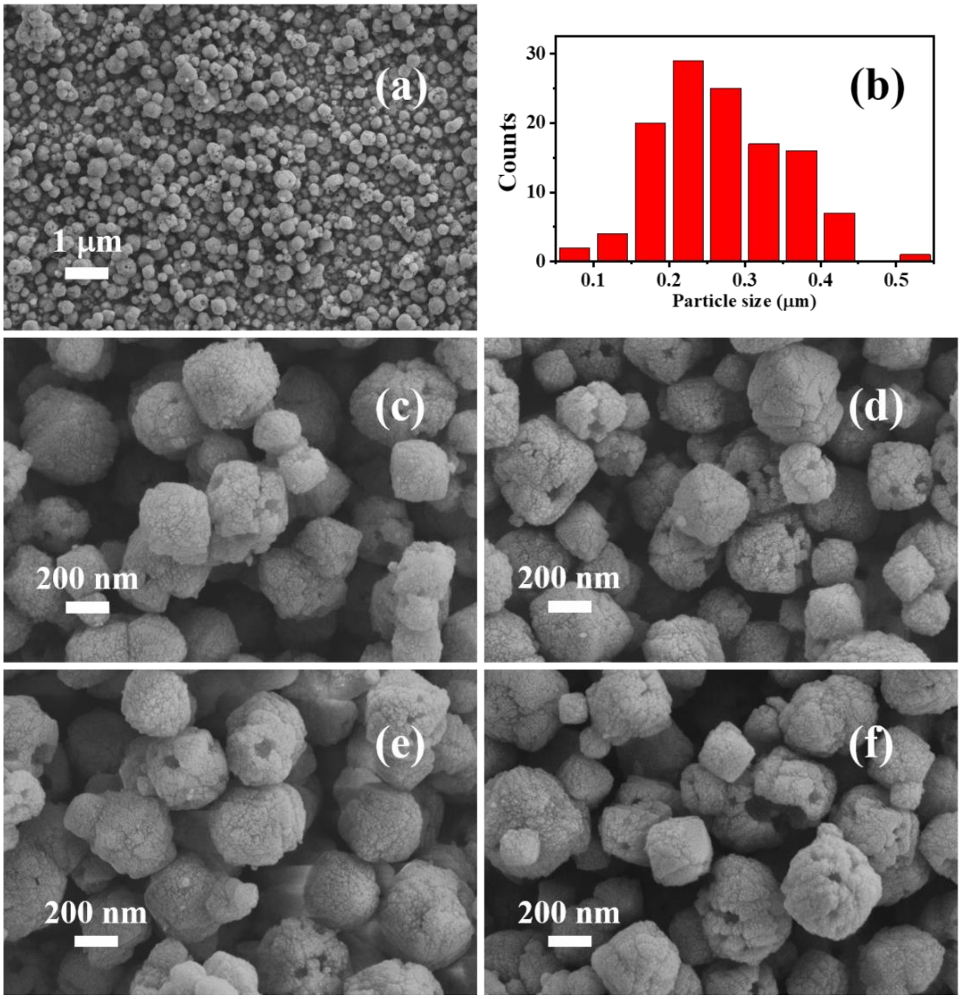

Figure S3. Scanning electron microscope (SEM) images of iron-exchanged BEA35 zeolites with various iron contents: (a) BEA35, (c) 0.52 Fe-BEA35, (d) 1.26 Fe-BEA35, (e) 1.61 Fe-BEA35, and (f) 2.36 Fe-BEA35; (b) the particle size distribution of BEA35 zeolites.

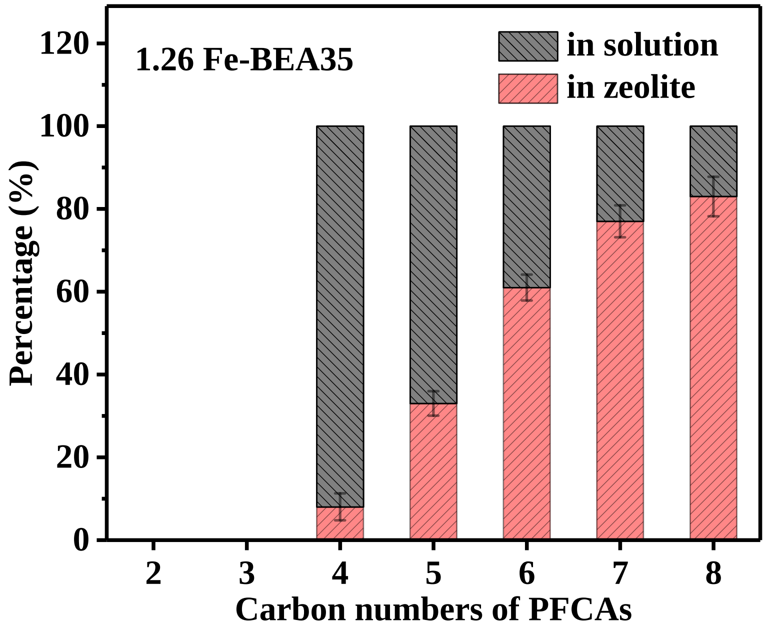

Figure S4. Distribution of PFCAs with various chain lengths between zeolite 1.26 Fe-BEA35 (X_{sorb}) and the aqueous phase (X_{free}) after 1 day of shaking. 1 g L^{-1} 1.26 Fe-BEA35, $C_{0,PFCAs}$ = 48 μM and pH_0 = 5. PFCAs with chains of 2 and 3 carbons did not show a significant sorption effect.

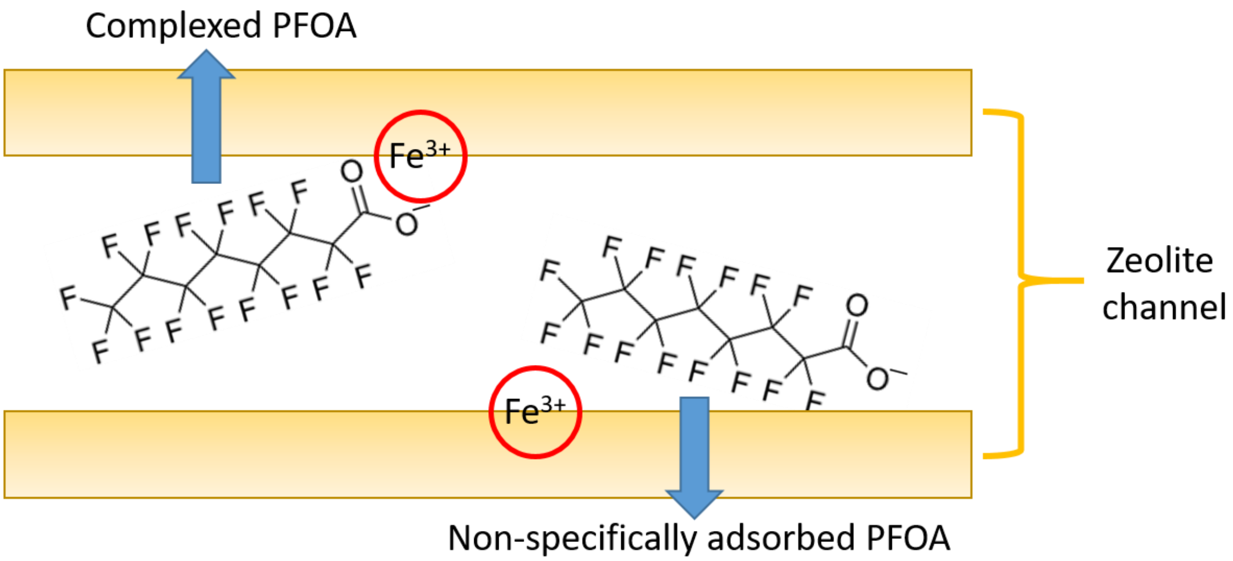

Figure S5. Schematic diagram of PFOA configurations on Fe-BEA35 with and without specific adsorption. The term “complexed PFOA” means the specifically adsorbed PFOA at ferric ions.

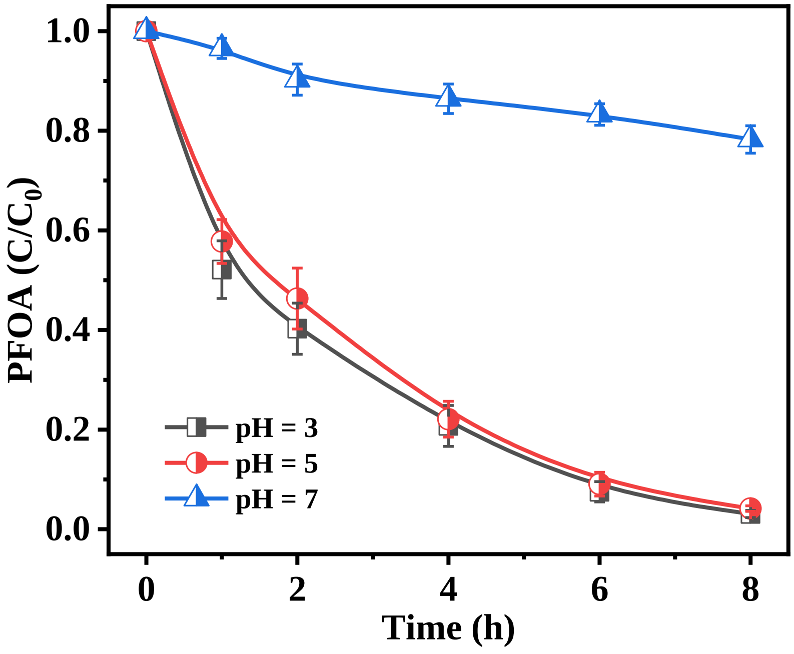

Figure S6. Photodegradation of PFOA by Fe-BEA35 zeolite at various initial pH values. 1 g L^{-1} 1.26 Fe-BEA35, $C_{0,\mathrm{PFOA}}$ = 48 μM. Error ranges stand for the standard deviations of the results from triplicate assays. Lines serve as guides for the eye.

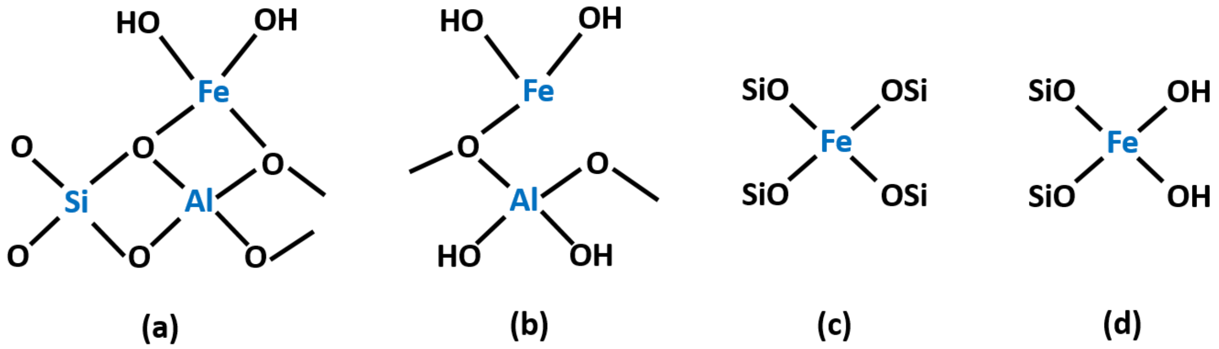

Figure S7. Schematic diagram of four isolated Fe species (as examples) in Fe-containing zeolites catalysts. (a) Fe species at cation exchange sites; (b) mono-nuclear Fe species coordinated to extra-framework Al(III); (c) framework Fe species; and (b) extra-framework Fe species tied to silicon hydroxyl nests. [12-14]

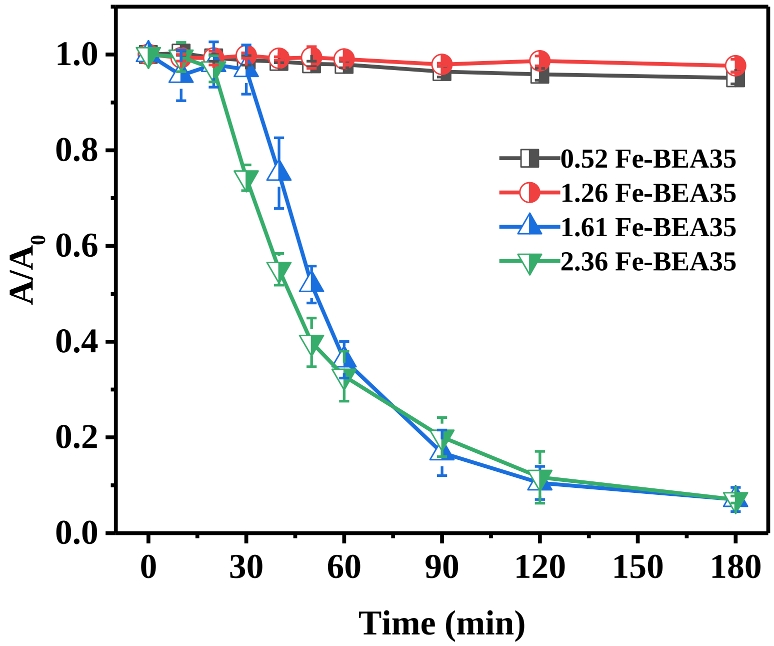

Figure S8. Stability of aqueous zeolite suspensions (2.5 g L^{-1} zeolite in 100 mM KNO_3, pH=3.5) determined by sedimentation analysis (continuous time-resolved measurement of relative absorbance A/A_0 at 725 nm at a depth of 20 mm below water level).

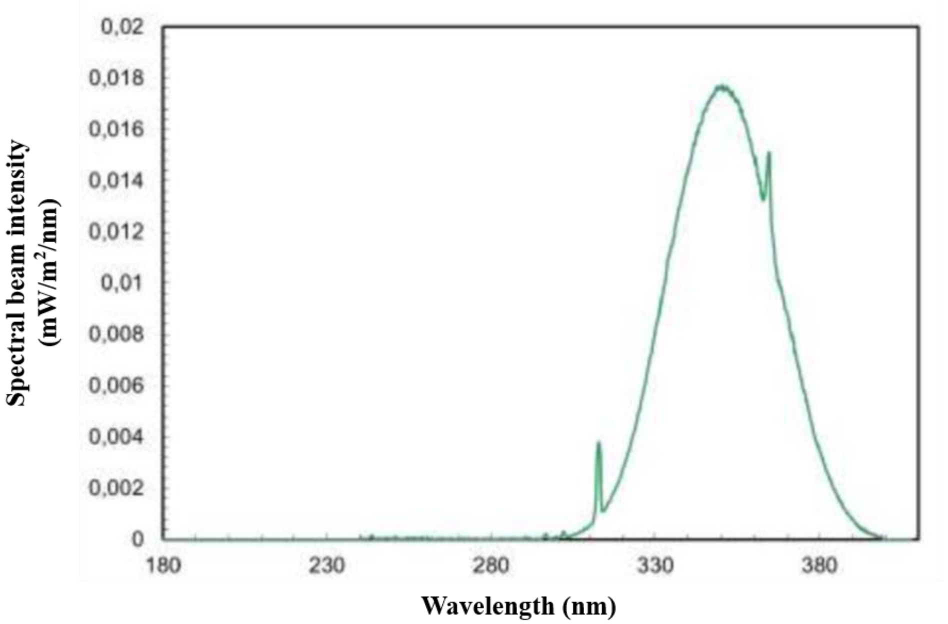

Figure S9. Spectral beam intensity of UV-A lamp. The intensity varies along with power of the lamp. The spectral curve was acquired from the UV lamp provider (https://www.herolab.de).

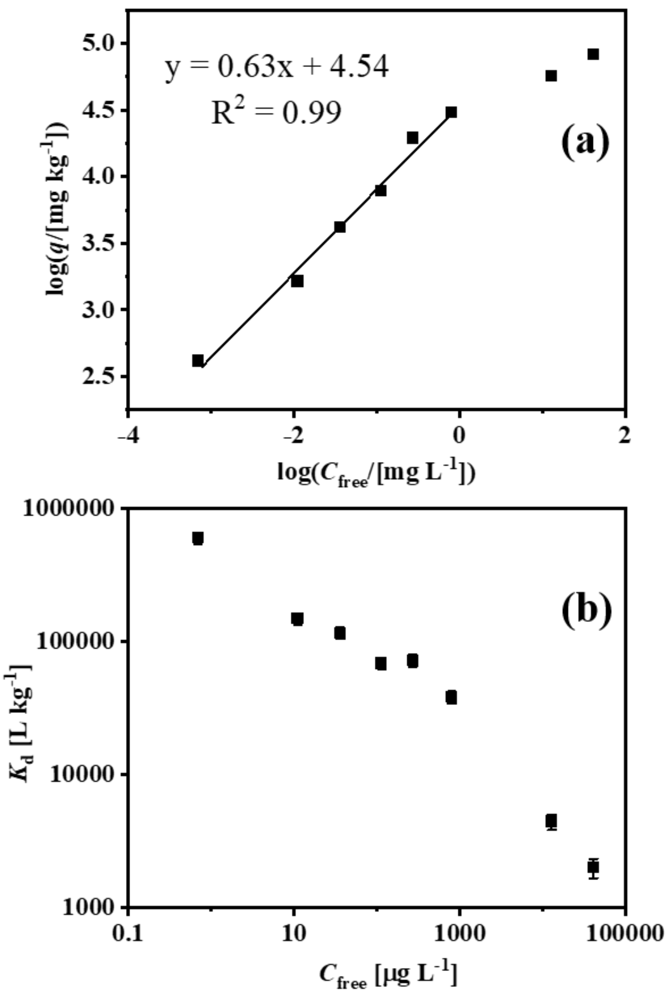

Figure S10. Adsorption isotherms (a) and single-point sorption coefficients as function of equilibrium aqueous phase PFOA concentration (b) for adsorption of PFOA on BEA35 zeolite in EPA standard soft water, pH 7.[5]

Langmuir isotherm fit for the adsorption of PFOA on BEA35 zeolite

The linearized form of the Langmuir isotherm (eq. S14) was applied to fit the adsorption equilibrium data. In contrast to the Freundlich isotherm, the fit was in this case better in the high loading range. In this way, the maximum loading (q_m = 7.7 wt.%) and Langmuir coefficient (K_L = 1100 L g^{-1}) were determined.

$$\frac{1}{q_e} = \frac{1}{q_m} + \frac{1}{q_m \cdot K_L \cdot C_w} \qquad \text{(S14)}$$

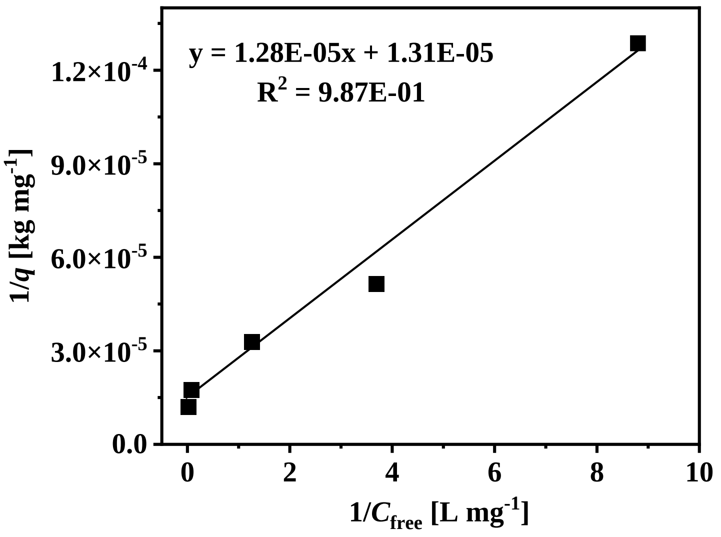

Figure S11. Adsorption isotherm for adsorption of PFOA on BEA35 zeolite in EPA standard soft water, pH = 7 fitted by the Langmuir equation (for data points with $q_m \geq 5$ g kg^{-1} or 0.5 wt.%)

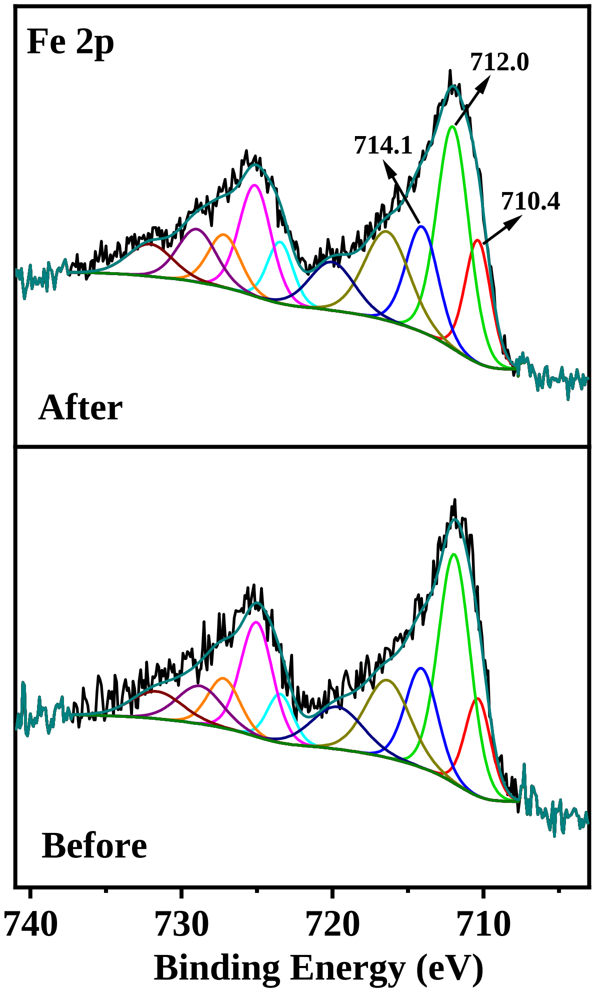

Figure S12. Fe 2p XPS spectra of 1.26 Fe-BEA35 before and after photodegradation of PFOA.

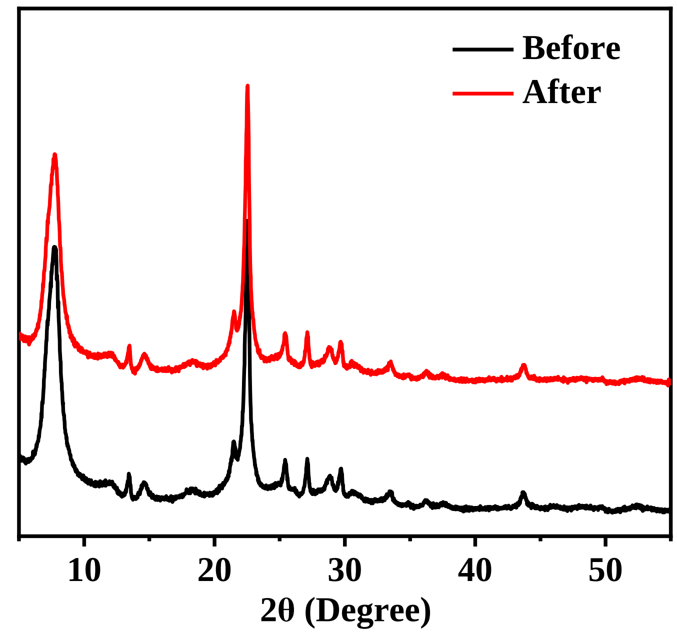

Figure S13. The X-ray powder diffraction pattern of 1.26 Fe-BEA35 before and after photodegradation of PFOA

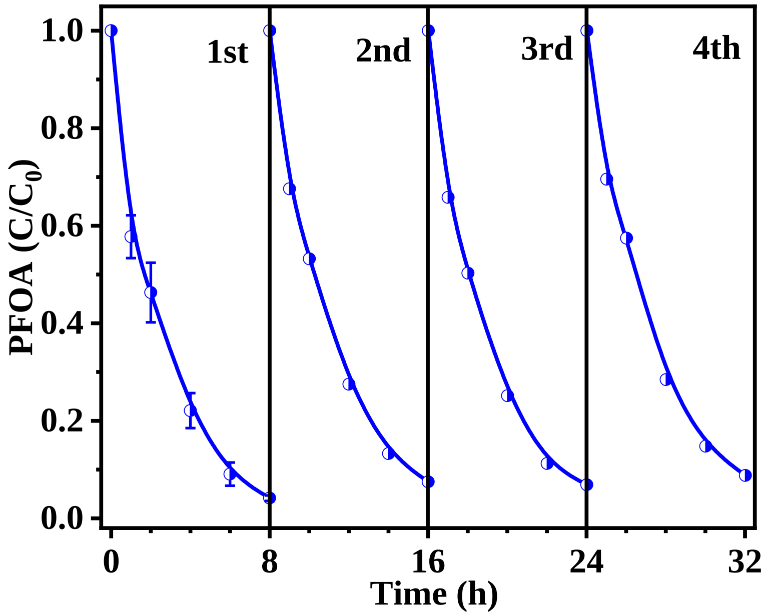

Figure S14. Reusability test of Fe-zeolite for PFOA degradation under UV-A irradiation. 1 g L^{-1} 1.26 Fe-BEA35, $C_{0,PFOA}$ = 48 µM each, pH_0 = 5. Lines serve as guides for the eye.

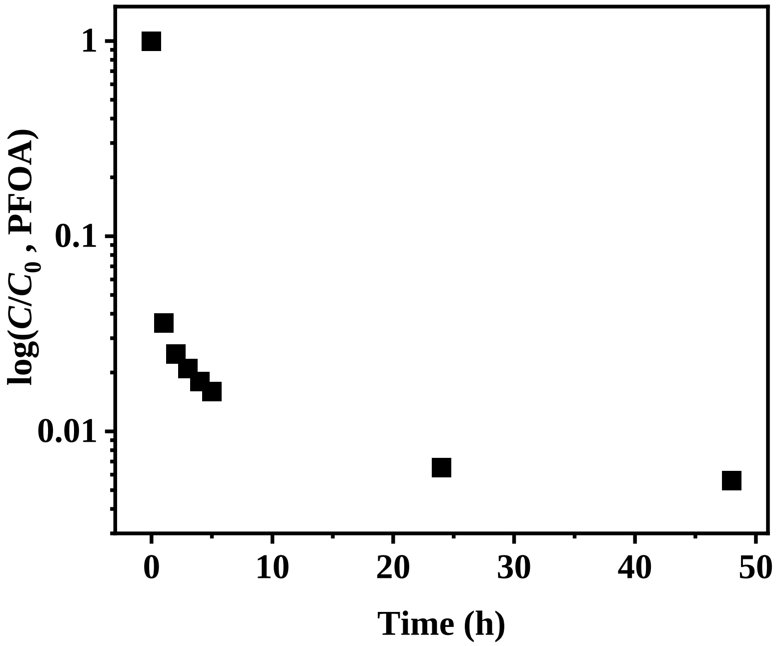

Figure S15: Kinetics of PFOA adsorption on Fe-BEA35 zeolite (10 g L^{-1} 1.26 Fe-BEA35, $C_{0,PFOA}$ = 48 μM)

Adsorption kinetics is biphasic with a fast initial part where >90% of PFOA are adsorbed and a slower part most likely due to slow diffusion of PFOA in the zeolite pore system with approach to equilibrium within 24 h.

References.

1. Gillies, G.; Raj, R.; Kopinke, F.-D.; Georgi, A., Suspension stability and mobility of Trap-Ox Fe-zeolites for in-situ nanoremediation. *Journal of Colloid and Interface Science* **2017,** *501*, 311-320.
2. Qian, L.; Georgi, A.; Gonzalez-Olmos, R.; Kopinke, F.-D., Degradation of perfluorooctanoic acid adsorbed on Fe-zeolites with molecular oxygen as oxidant under UV-A irradiation. *Applied Catalysis B: Environmental* **2020,** *278*, 119283.
3. Qian, L.; Kopinke, F.-D.; Georgi, A., Photodegradation of Perfluorooctanesulfonic Acid on Fe-Zeolites in Water. *Environmental Science & Technology* **2021,** *55*, (1), 614-622.
4. Qian, L.; Kopinke, F.-D.; Scherzer, T.; Griebel, J.; Georgi, A., Enhanced degradation of perfluorooctanoic acid by heat-activated persulfate in the presence of zeolites. *Chemical Engineering Journal* **2022,** *429*, 132500.
5. Agency, U. S. E. P., EPA-821-R-02-012: Methods for Measuring the Acute Toxicity of Effluents and Receiving Waters to Freshwater and Marine Organisms. In 2002.
6. Vercammen, J.; De Vos, D., Reply to Comment on "Highly Selective Removal of Perfluorinated Contaminants by Adsorption on All-Silica Zeolite Beta". *Angewandte Chemie International Edition* **2021,** *60*, (25), 13710-13711.
7. Aumeier, B. M.; Georgi, A.; Saeidi, N.; Sigmund, G., Is sorption technology fit for the removal of persistent and mobile organic contaminants from water? *Science of The Total Environment* **2023,** *880*, 163343.
8. Kutsuna, S.; Hori, H., Rate constants for aqueous-phase reactions of SO4- with C2F5C(O)O- and C3F7C(O)O- at 298 K. *Int. J. Chem. Kinet.* **2007,** *39*, (5), 276-288.
9. Wang, Y.; Zhang, P.; Pan, G.; Chen, H., Ferric ion mediated photochemical decomposition of perfluorooctanoic acid (PFOA) by 254 nm UV light. *J. Hazard. Mater.* **2008,** *160*, (1), 181-186.
10. Jin, L.; Zhang, P.; Shao, T.; Zhao, S., Ferric ion mediated photodecomposition of aqueous perfluorooctane sulfonate (PFOS) under UV irradiation and its mechanism. *J. Hazard. Mater.* **2014,** *271*, 9-15.
11. Lutze, H. V.; Brekenfeld, J.; Naumov, S.; von Sonntag, C.; Schmidt, T. C., Degradation of perfluorinated compounds by sulfate radicals–New mechanistic aspects and economical considerations. *Water Res.* **2018,** *129*, 509-519.
12. Xia, H.; Hu, H.; Xu, S.; Xiao, K.; Zuo, S., Catalytic conversion of glucose to 5-hydroxymethyfural over Fe/β zeolites with extra-framework isolated Fe species in a biphasic reaction system. *Biomass and Bioenergy* **2018,** *108*, 426-432.
13. Yasumura, S.; Qian, Y.; Kato, T.; Mine, S.; Toyao, T.; Maeno, Z.; Shimizu, K.-i., In Situ/Operando Spectroscopic Studies on the NH3–SCR Mechanism over Fe–Zeolites. *ACS Catalysis* **2022,** *12*, (16), 9983-9993.
14. Zhang, J.; Tang, X.; Yi, H.; Yu, Q.; Zhang, Y.; Wei, J.; Yuan, Y., Synthesis, characterization and application of Fe-zeolite: A review. *Applied Catalysis A: General* **2022,** *630*, 118467.
15. Gurgul, J.; Łątka, K.; Hnat, I.; Rynkowski, J.; Dzwigaj, S., Identification of iron species in FeSiBEA by DR UV–vis, XPS and Mössbauer spectroscopy: Influence of Fe content. *Microporous and Mesoporous Materials* **2013,** *168*, 1-6.
16. Tan, P., Active phase, catalytic activity, and induction period of Fe/zeolite material in nonoxidative aromatization of methane. *Journal of Catalysis* **2016,** *338*, 21-29.
